# Phenolic compounds weaken the impact of drought on soil enzyme activity in global wetlands

**DOI:** 10.3389/fmicb.2024.1372866

**Published:** 2024-03-08

**Authors:** Tong Li, Leming Ge, Ruotong Zhao, Changhui Peng, Xiaolu Zhou, Peng Li, Zelin Liu, Hanxiong Song, Jiayi Tang, Cicheng Zhang, Quan Li, Meng Wang, Ziying Zou

**Affiliations:** ^1^School of Geographic Sciences, Hunan Normal University, Changsha, China; ^2^Key Laboratory of Geographical Processes and Ecological Security in Changbai Mountains, Ministry of Education, School of Geographical Sciences, Northeast Normal University, Changchun, China; ^3^Department of Biology Science, Institute of Environment Sciences, University of Quebec at Montreal, Montreal, QC, Canada; ^4^State Key Laboratory of Subtropical Silviculture, Zhejiang A&F University, Hangzhou, China; ^5^State Environmental Protection Key Laboratory of Wetland Ecology and Vegetation Restoration, Institute for Peat and Mire Research, Northeast Normal University, Changchun, China

**Keywords:** drought, wetland ecosystems, peat, resilience, polyphenols, enzymatic sensitivity

## Abstract

Soil enzymes play a central role in carbon and nutrient cycling, and their activities can be affected by drought-induced oxygen exposure. However, a systematic global estimate of enzyme sensitivity to drought in wetlands is still lacking. Through a meta-analysis of 55 studies comprising 761 paired observations, this study found that phosphorus-related enzyme activity increased by 38% as result of drought in wetlands, while the majority of other soil enzyme activities remained stable. The expansion of vascular plants under long-term drought significantly promoted the accumulation of phenolic compounds. Using a 2-week incubation experiment with phenol supplementation, we found that phosphorus-related enzyme could tolerate higher biotoxicity of phenolic compounds than other enzymes. Moreover, a long-term (35 years) drainage experiment in a northern peatland in China confirmed that the increased phenolic concentration in surface layer resulting from a shift in vegetation composition inhibited the increase in enzyme activities caused by rising oxygen availability, except for phosphorus-related enzyme. Overall, these results demonstrate the complex and resilient nature of wetland ecosystems, with soil enzymes showing a high degree of adaptation to drought conditions. These new insights could help evaluate the impact of drought on future wetland ecosystem services and provide a theoretical foundation for the remediation of degraded wetlands.

## 1 Introduction

Drought resulting from climate and land-use changes poses a major challenge to the storage of soil carbon and nutrients in terrestrial ecosystems (Berdugo et al., 2020; Muller and Bahn, 2022). This water scarcity induces xylem cavitation and reduces stomatal conductance, thereby impeding plants from reaching their full photosynthetic potential (Choat et al., 2018; Guo et al., 2021). Several studies have observed a decrease in primary production during severe drought events (Angert et al., 2005; Doughty et al., 2015; Liu et al., 2015). In addition to decreasing plant-derived organic inputs, drought can pose a threat to soil carbon and nutrient sequestration through increased decomposition (Haugwitz et al., 2016; Tsiafouli et al., 2018). Water availability plays a crucial role in shaping the function of microbial decomposers (Zhang et al., 2023). A sharp decline in the soil carbon sink function has been reported when water availability (i.e., aridity index) dropped below 0.25, due to diminished microbial diversity and altered nutrient mineralization (Zhang et al., 2023).

The quantification of soil extracellular enzyme activities (EEAs) provides valuable insights into evaluating the impact of drought on soil biogeochemical cycles. Soil enzymes, produced and released by microorganisms and plant roots, play a central role in promoting organic matter decomposition (Moorhead and Sinsabaugh, 2006; Zuccarini et al., 2023). C-, N-, and P-related EEAs, being substrate-specific, serve as crucial indicators of the energy or nutrient demand of microorganisms and plants (Cui et al., 2021). Soil enzymes are highly sensitive to drought (Brockett et al., 2012; Deng et al., 2021). Moreover, the low cost and high technical feasibility associated with measuring soil EEAs have encouraged numerous primary studies (Zuccarini et al., 2023), enabling the incorporation of these findings into a meta-analysis that synthesizes the responses of soil EEAs to drought at regional and global scales.

Several systematic reviews and meta-analyses have synthesized the impact of drought on soil EEAs in typical terrestrial ecosystems (Ren et al., 2017; Xiao et al., 2018; Deng et al., 2021; Margalef et al., 2021). However, some habitat-specific ecosystems, such as wetland, tundra, and desert ecosystems, are often overlooked due to their small sample size in these syntheses. Wetlands, in particular, serve as important carbon pools, storing ~30% of global terrestrial carbon (Schlesinger and Bernhardt, 2020). This massive carbon storage is mainly attributed to an imbalance between photosynthetic production and enzyme-mediated decomposition under seasonal or perennial waterlogged conditions (Wang et al., 2015). The responses of soil EEAs to drought in wetlands diverge from those observed in dryland ecosystems. In wetlands, waterlogged soil to oxygen, thereby promoting soil EEAs (Henry, 2012). On the contrary, in forests and grasslands, water scarcity can suppress soil EEAs by reducing the enzyme diffusion rate and decreasing microbial production (Zuccarini et al., 2023).

In wetlands, there is also a competing mechanism that counteracts the positive feedback between oxygen-enhanced soil EEAs and organic matter decomposition during drought. This mechanism is centered around enzyme-inhibiting phenolic compounds (Freeman et al., 2001; Wang et al., 2015). Phenolic compounds are important secondary metabolites in plants and fungi, and they can also be leached from litter (Wan et al., 2018; Fenner and Freeman, 2020). The accumulation of phenolic compounds can limit nutrient cycling and protect soil carbon by binding proteins, thus inhibiting hydrolytic enzyme activities via oxidative degradation and biotoxicity (Fenner and Freeman, 2020). As an external driver regulating plant growth, long-term drought can alter plant metabolism and even trigger the adaptive succession of wetlands, resulting in the expansion of high-phenolic woody plants (Laiho et al., 2003; Talbot et al., 2010). This leads to the further accumulation of phenolic compounds and therefore inhibit soil EEAs; consequently, soil organic matter is stabilized regardless of the oxygen status (Wang et al., 2015; Li et al., 2020). However, under the antagonistic action of these two mechanisms, the global patterns of soil EEAs in wetlands during drought remain under-investigated, and the future fate of carbon and nutrients stored in wetlands under the changing climate remains unclear.

The objective of this study was to use meta-analysis coupled with incubation and drainage experiments to evaluate the intricate impacts of drought on soil EEAs in wetlands. First, a meta-analysis was conducted using 761 paired observations from 55 peer-reviewed studies, aiming to quantitatively synthesize the global response patterns of soil EEAs to drought in wetlands and provide a related global dataset for the first time. This meta-analysis encompassed three categories of hydrolytic enzymes (C-, N-, and P-related enzymes) and one oxidase, and it also included various soil chemical properties such as phenolic compounds, as well as carbon, nitrogen, and phosphorus concentrations. Second, building on the insights gained from the meta-analysis, a 2-week incubation experiment involving the addition of phenol was subsequently conducted to investigate the regulation of phenolic concentration on soil EEAs under aerobic conditions. Lastly, the responses of key hydrolytic enzyme activities to long-term, *in-situ* drainage were examined and compared at two levels of phenolic compounds in a minerotrophic peatland in northeastern China. We intended to test the following two hypotheses: (1) drought would increase the oxidase activity in wetlands; (2) the increased phenolic compounds input after long-term drought would inhibit the activities of hydrolytic enzymes.

## 2 Data and methodology

### 2.1 Literature data collection

**Figure 1:**
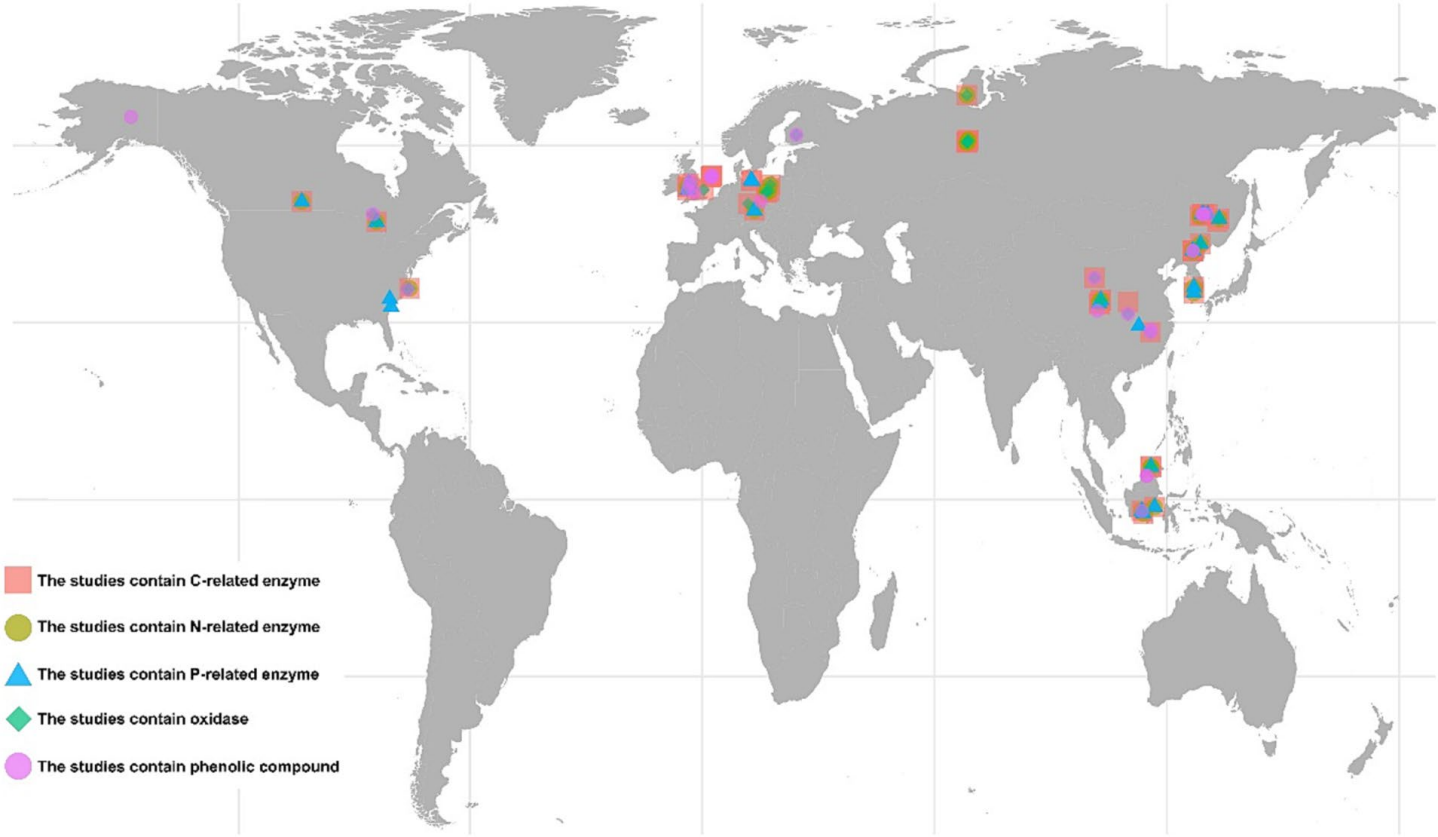
Geographical location of the studies included in the meta-analysis. The pink squares represent the 35 studies that contain C-related enzymes. The red squares represent the 35 studies that contain C-related enzymes. The green circles represent the 22 studies that contain N-related enzymes. The blue triangles represent the 26 studies that contain P-related enzymes. The light green rhombuses represent the 28 studies that contain oxidases. The pink circles represent the 11 studies that phenolic compounds. The studies may involve in multiple sampling sites.

The ISI Web of Science^1^ and China National Knowledge Infrastructure^2^ were searched for peer-reviewed papers (last searched on October 31, 2022). The complete list of search strings is shown in Supplementary Table S1. The following criteria were adopted to select related studies: (1) only studies conducted in natural wetlands were included; (2) all types of drought (such as drainage and precipitation deficit) were accepted except warming; (3) the means, samples sizes, standard errors, or deviations could be directly obtained from the text, tables, figures, or appendices of the publications; (4) only data from the surface soil layer were included; and (5) the treatments combining drought with other interventions were not included. Additionally, for the phenolic compounds, the included studies used diverse physical and chemical methods to determine their concentrations or relative amounts. This precluded the further classification of phenolic compounds based on their chemical composition. A flowchart of the process and outcome of the literature search is provided in Supplementary Figure S1. The final dataset contained 761 paired observations derived from 55 papers (Figure 1).

The dataset included 12 variables: (1) one C-related enzyme (subdivided into β-1,4-glucosidase, β-1,4-xylosidase, invertase, β-D-cellobiohydrolase, α-1,4-glucosidase, invertase, and amylase); (2) one N-related enzyme (subdivided into β-1,4-N-acetylglucosaminidase, leucine amino peptidase, chitinase, and urease); (3) one P-related enzyme (phosphatase); (4) one oxidase (subdivided into phenol oxidase and peroxidase); (5) one category of phenolic compound; (6) seven soil properties, namely, the total carbon, total organic carbon, dissolved organic carbon, total nitrogen, total phosphorus, pH, and microbial biomass carbon.

### 2.2 Peat incubation with phenol supplementation

In order to test the sensitivities of soil EEAs to phenolic toxicity, we conducted the following incubation experiment. Peat samples for the incubation experiment were collected from the Hani Peatland (42°13′N, 126°31′E) in northeastern China. This region exhibits a continental monsoon climate within a cold temperate region, with a mean annual precipitation of 757–930 mm and a mean annual temperature of 2.5–3.6°C (Bu et al., 2011). The peat reserves exceed 1.2 × 10^7^ t and are still in the development stage. Hani Peatland is dominated by *Sphagnum palustre* L., *Sphagnum magellanicum* Brid., *Eriophorum polystachion* L., and *Carex lasiocarpa* Ehrh., in addition to shrubs, including *Betula ovalifolia* Rupr. and *Vaccinium uliginosum* L. (Bu et al., 2017). Samples were collected in small patches over a pristine area of 100 m^2^ to form a composite sample (> 2 kg). All sampling sites had a similar water-table level, above-ground plant species composition, and microtopography. Living mosses were removed from samples, and the remainder of the sample was placed in a sterile plastic bag and transported back to the laboratory on ice.

The incubation setup was adapted from Dunn and Freeman (2017) with triplicates. Peat samples were first placed on multiple layers of absorbent paper to remove excess soil water. Subsequently, 24 g peat was added to a 100-mL sterilized glass bottle, which was sealed using a hydrophobic fluoropore membrane with a 0.22-μm aperture size to prevent possible microbial interfaces from the air and ensure free gas exchange. All bottled peat samples were activated at room temperature (about 25°C) for 1 day before the incubation experiment. Phenol was dissolved in sterilized deionized water and successively diluted into six concentrations of 0, 4, 8, 12, and 20 mg phenol L^−1^. The 24 mL phenol solution was added to the glass bottle to form a series of peat–solution slurries with different phenol concentrations. The incubation experiment was maintained in an incubator at 28°C and 50% relative humidity. Peat in the bottles was destructively sampled for each treatment on days 0, 1, 3, 5, 7, and 14.

After each sampling, we measured the activities of β-D-glucosidase (BDG), β-1,4-N-acetylglucosaminidase (NAG), and phosphatase (PHO) in the peat samples using a method adapted from Dunn et al. (2014). Briefly, these enzyme activities were measured using the microplate-based fluorescence method at an excitation wavelength of 330 nm and an emission wavelength of 450 nm on a multi-function microplate reader (M200PRO, TECAN, Switzerland). The standard was prepared with 4-methylumbelliferone (MUF) sodium salt. For the measurement of each enzyme, 7 mL of the substrate labeled with 4-MUF and 1 g of moist peat were placed together in a stomacher bag and incubated at field temperature for 45 min for PHO or 60 min for the other enzymes.

### 2.3 Long-term drainage experiment

In order to investigate the inhibitory effects of phenolic compounds on soil EEAs during drought conditions, we conducted the following long-term drainage experiment. In August 2023, surface peat samples were collected from a long-term drainage area of Baijianghe Peatland (42°09′N, 126°44′E) in northeast China. Because the Baijianghe and Hani peatlands are about 20 km apart, they have nearly identical climate characteristics. At this long-term drainage site, the surface peat is entirely exposed to the air. The moisture content of surface peat in the drained and undrained areas is approximately 1 and 4.5 g water g^−1^ dry peat, respectively. Part of the peatland was ditched and drained for agriculture in 1987. To date, the long-term drainage period of about 35 years has facilitated the establishment of a large number of shrubs. Many *Sphagnum* hummocks are encroached upon by clusters of shrubs, such as *Potentilla fruticosa* L., *Betula ovalifolia* Rupr., and *Rhododendron lapponicum* (L.) Wahlenb. High-phenolic litter from shrubs increases the concentration of phenolic compounds in the microtopography (Li et al., 2020).

An improved Folin–Ciocalteu method from Box (1983) was first used to determine the concentration of soil phenolic compounds at multiple random sites in the drainage area. Briefly, phenol was used to prepare the standard over a range of 0–45 mg/L. To obtain the concentration of phenolic compounds, 1 g of peat was placed in a centrifuge tube with a 0.45-μm filter insert (Costar spin-X, Cole-Parmer) and centrifuged at 10,000 rpm for 5 min. The mixture (250 μL filtrate +12.5 μL Folin’s reagent +37.5 μL Na_2_CO_3_ solution) was allowed to incubate in a 96-well clear microplate for 1.5 h. Subsequently, the absorbance was measured at 750 nm on a full-wavelength microplate reader (MULTISKAN GO, THERMO FISHER, Finland), and then converted into a concentration value. Based on the above results, two sites in the drainage area with significantly different concentrations of phenolic compounds were identified. The activities of BDG, NAG, and PHO in these two sites were measured using the method mentioned above in quadruplicate.

### 2.4 Statistical analysis

A meta-analysis was performed on the literature dataset. The method for meta-analysis was adopted by Hedges et al. (1999). The log response ratio (*RR*) was calculated as a measure of effect size for each observation using Equation (1).


(1) $$RR=\ln\left(X_{t}/X_{c}\right)$$

where *X_t_* and *X_c_* are the mean of the treatment and control groups, respectively.

The variance (*v*) of *RR* was calculated using Equation (2).


(2) $$v=S_{t}^{2}/N_{t}X_{t}^{2}+S_{c}^{2}/N_{c}X_{c}^{2}$$

where *S*, *N*, and *X* are the standard deviation, sample size, and mean, respectively; and the subscripts *t* and *c* represent the treatment and the control group, respectively.

**Figure 2:**
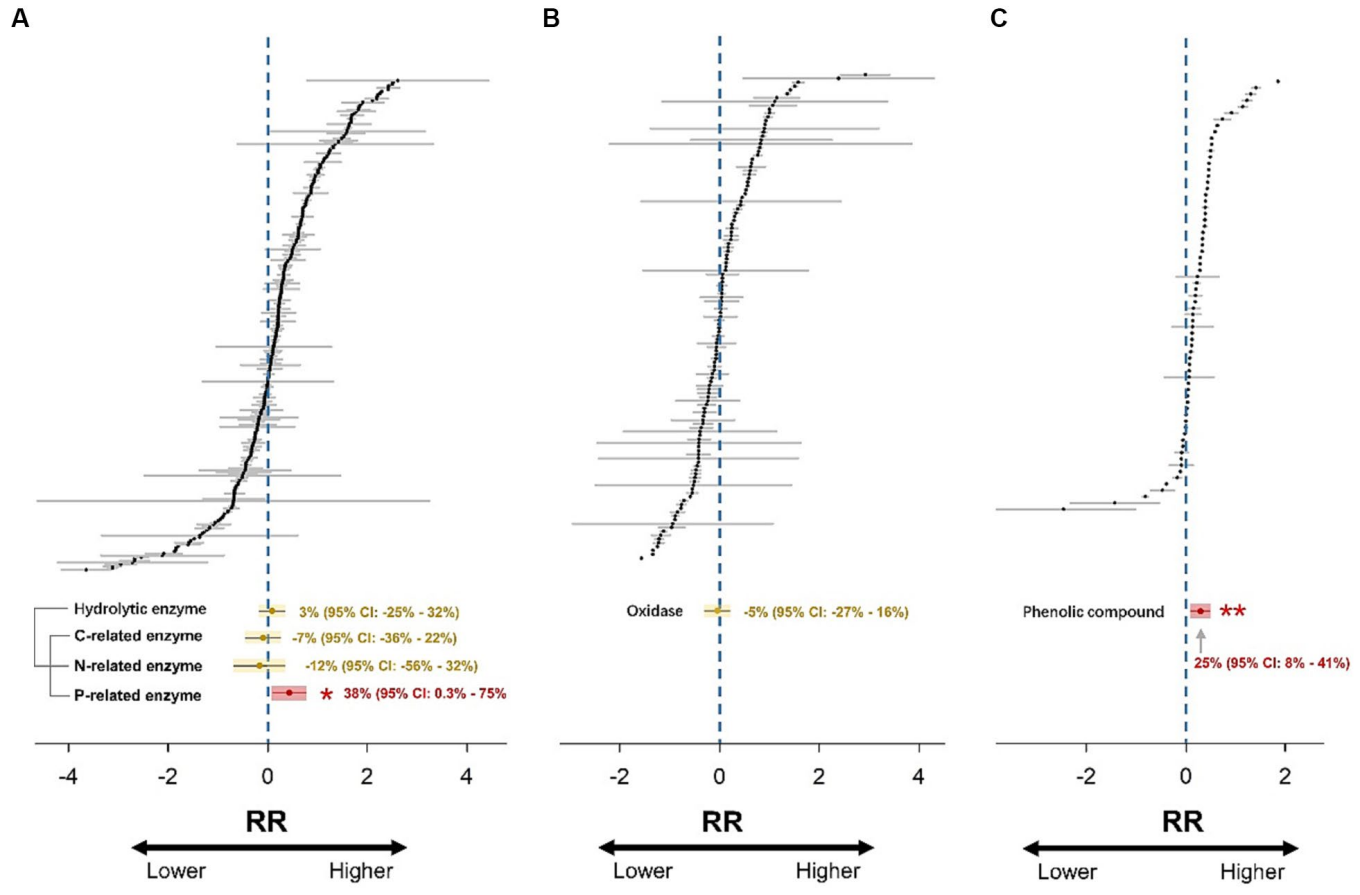
Forest plots of 271, 127, and 69 effect size estimates for hydrolytic enzymes **(A)**, oxidases **(B)**, and phenolic compounds **(C)**. RR represents the response ratio (effect size). Gray lines represent 95% confidence intervals. Overall weighted mean effect size estimates are indicated by yellow (non-significant response) or red (significant response) dots with yellow or red shadows. The 95% confidence intervals of the weighted mean effect size are indicated by yellow (non-significant response) or red (significant response) lines. The effect of drought is significant if the confidence interval of the mean effect does not include zero.

A hierarchical mixed-effects meta-analysis model with random effects was run to calculate the weighted effect size (*RR_++_*) using the “*metafor*” package in R (Viechtbauer, 2010). This model took into account the hierarchical dependencies of the data in cases where multiple observations were obtained from the same study; namely, observations from the same study were considered independent (Tuck et al., 2014). The random effect structure was “random = list (~1|Study).” The number of iterations was set to 999, and the 95% confidence intervals (CIs) were reported. The treatment effect was considered significant (*p* < 0.05) if the 95% CI did not overlap with zero (Figure 2).

The present study evaluated the heterogeneity of effect sizes using *Q* tests (*Q_t_*) to determine whether the models could explain a significant amount of variation (Supplementary Table S2). As a result, there was significant residual heterogeneity in the meta-analysis for the C-related enzyme dataset (*Q_t_* = 9,210, *p* < 0.001), N-related enzyme dataset (*Q_t_* = 12,582, *p* < 0.001), P-related enzyme dataset (*Q_t_* = 49,687, *p* < 0.001), oxidase dataset (*Q_t_* = 3,709, *p* < 0.001), and phenolic compound dataset (*Q_t_* = 962, *p* < 0.001). Thus, data were categorized into the following four subgroups: “dominant plant” (including moss, sedge, and wood), “experiment duration” (short- (≤3 years), medium- (3–5 years), and long-term (≥5 years) experiments), “experimental type” (field, microcosm, and incubation), and “peat condition” (yes or no). The data of these four subgroups were used for subgroup analysis to explain the significant residual heterogeneity of RR_++_. The *Q_m_*-statistic test (*Q_m_*) was used to determine whether the classification type explained any significant heterogeneity in the data, and the accompanying *Qe* was the residual error variance. The rank correlation between individual effect size and standard error was used to test publican bias (Supplementary Table S3).

For the incubation and drainage experiments, the data normality and the homogeneity of variance were examined with Shapiro–Wilk and Levene’s tests, respectively. One-way analysis of variance (ANOVA) was used to examine the effect of phenol addition on enzyme activities, and multiple comparisons were conducted using Tukey’s post-hoc tests. A two-sample *t*-test was used to compare enzyme activities between the drainage treatment and control samples for the results of the drainage experiment. Statistical analyses were considered to be significant at *p* < 0.05.

## 3 Results

### 3.1 Responses of enzyme activities and phenolic compounds in global wetlands

The results showed that hydrolytic enzyme activity did not change significantly after drought (Figure 2A). Subsequently, the hydrolytic enzyme dataset was partitioned into three sub-datasets of C-, N-, and P-related enzymes. The activities of C- and N-related enzymes still did not change significantly, while the P-related enzyme activity increased significantly by 38% due to drought (*p* < 0.05; Figure 2A). The oxidase activity did not change significantly in response to drought (Figure 2B). Moreover, drought promoted the accumulation of phenolic compounds by 25% (*p* < 0.01) in global wetlands (Figure 2C).

**Figure 3:**
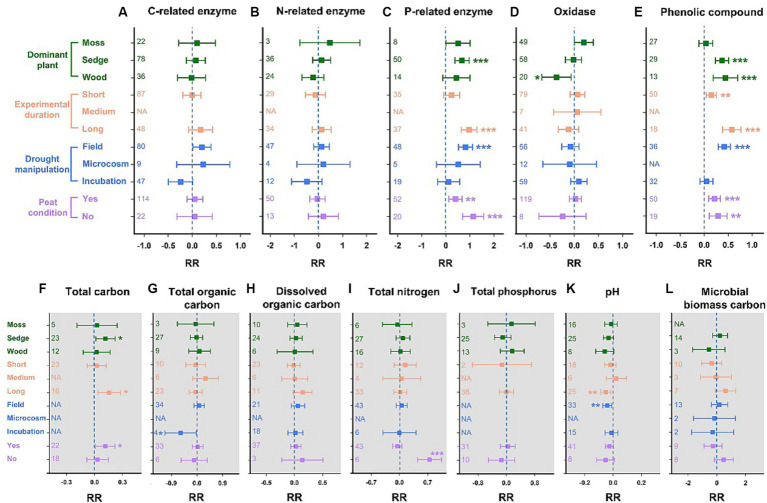
Changes in the activities of soil enzymes and the concentration of phenolic compounds, as well as soil properties for different levels of dominant plants, experimental durations, the type of drought manipulation, and peat conditions. **(A)** Carbon-related enzymes, **(B)** nitrogen-related enzymes, **(C)** phosphorus-related enzymes, **(D)** oxidases, and **(E)** phenolic compounds. **(F)** Total carbon, **(G)** total organic carbon, **(H)** dissolved organic carbon, **(I)** total nitrogen, **(J)** total phosphorus, **(K)** pH, and **(L)** microbial biomass carbon. RR represents the weighted response ratio. The number of effect sizes is shown between brackets. Error bars represent 95% confidence intervals. The effect of drought is significant if the confidence interval of the mean effect does not include zero.

The C- and N-related enzyme activities remained stable in all subgroups (Figures 3A,B). A significant increase in P-related enzyme activities in sedge-dominated wetlands was observed (67%, *p* < 0.001; Figure 3C). Long-term drought significantly promoted P-related enzyme activity by 97% (*p* < 0.001), while short-term drought had no effect. Additionally, the P-related enzyme activity data obtained from field *in-situ* hydrologic manipulation showed a significant increase of 80% (*p* < 0.001). Whether soil was rich in peat did not influence the positive feedback between P-related enzyme activity and drought (peat conditions: +40%, *p* < 0.01; no-peat conditions: +113%, *p* < 0.001). Oxidase activities exhibited no significant responses to drought in all subgroups, except for in wood-dominated wetlands, where a significant decrease of 37% was observed (*p* < 0.05; Figure 3D). The phenolic compounds increased by 37% (*p* < 0.001) and 44% (*p* < 0.001) due to drought in the subgroups of sedge- and wood-dominated wetlands, respectively (Figure 3E). Short- and long-term drought significantly increased the concentrations of phenolic compounds by 15% (*p* < 0.01) and 58% (*p* < 0.001), respectively. Similar to the results for P-related enzyme activity, the *in-situ* drought experiment in the field could easily promote the accumulation of phenolic compounds (+42%, *p* < 0.001), and peat conditions in soil did not affect the positive response of phenolic compounds to drought (peat conditions: +22%, *p* < 0.001; no-peat conditions: +29%, *p* < 0.01).

### 3.2 Responses of soil properties in global wetlands

Overall, drought had a minor impact on wetland soil properties. For soil total carbon, the results indicated that drought significantly increased its concentration by 12 and 11% in sedge-dominated wetlands and peatlands, respectively (Figure 3F). Moreover, long-term drought has the potential to lead to higher soil carbon (+16%, *p* < 0.05). However, the total organic carbon, dissolved organic carbon, and microbial biomass carbon did not change remarkably during drought (Figures 3G,H,L). Drought significantly promoted the accumulation of total nitrogen in wetlands without peat (+75%, *p* < 0.001); however, it had no effect in other subgroups (Figure 3I). Analogously, no significant influence of drought was found on the soil’s total phosphorus, even after considering multiple scenarios (Figure 3J). Among the three drought durations, long-term drought significantly decreased the soil pH (−5%, *p* < 0.01; Figure 3K). Similarly, drought experiments in the field could significantly increase soil acidification (pH: −4%, *p* < 0.01).

### 3.3 Effects of phenolic concentration under drainage

**Figure 4:**
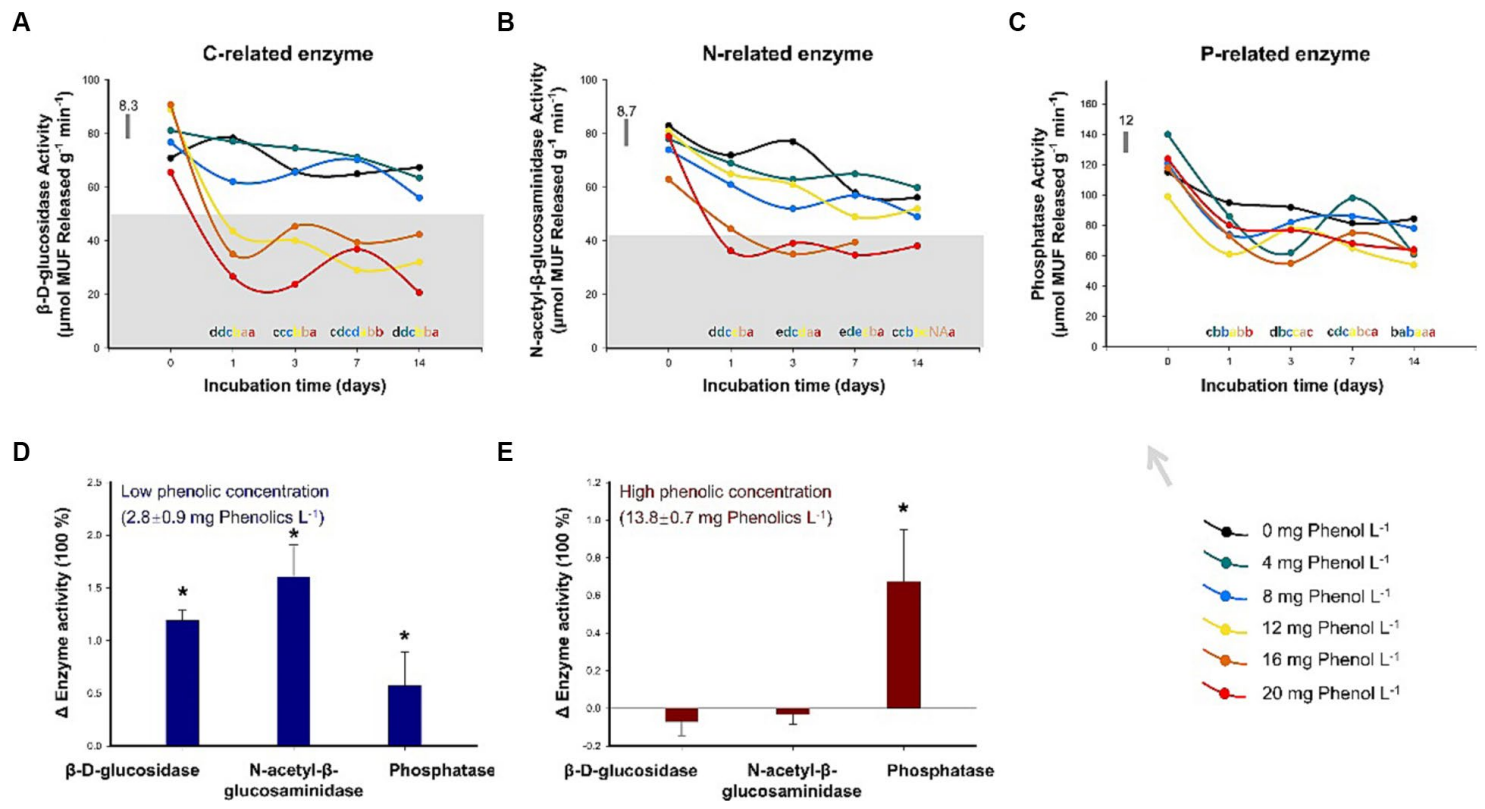
Sensitivity of key hydrolytic enzyme activities to different concentrations of phenolic compounds. The activities of β-D-glucosidase **(A)**, β-1,4-N-acetylglucosaminidase **(B)**, and phosphatases **(C)** were assessed over two weeks with the addition of phenol solutions at varying concentrations (*n* = 3). The activities of β-D-glucosidase, β-1,4-N-acetylglucosaminidase, and phosphatases after 35-year drainage in sites characterized by low **(D)** and high **(E)** concentrations of phenolic compounds in Baijianghe Peatland, expressed as the mean treatment triplicates relative to the control plots. In **(A–C)**, different lowercase letters represent significant differences (*p* < 0.05) among varying phenol concentrations with the same duration of incubation; the horizontal bar represents the average standard deviation among the difference between the treated and control triplicate plot samples. In **(D,E)**, an asterisk (*) represents significant differences (*p* < 0.05) between treated and control plots; error bars indicate standard errors.

For the incubation experiment, the addition of phenol generally resulted in a decrease in BDG and NAG activities, but the inhibitory effect of phenol on PHO was not observed. As shown in Figure 4A, the soil BDG activity was significantly inhibited when phenol addition exceeded 4 mg phenol L^−1^ following 1 day of incubation (*p* < 0.05). In the subsequent observations on days 3, 7, and 14, a high concentration of phenol (≥ 12 mg phenol L^−1^) consistently maintained the BAG activity at a lower level relative to the control (*p* < 0.05). A similar trend was found in NAG. After 1 day, the NAG activities significantly decreased by 15, 10, 38, and 50% relative to the control in the treatments with the addition of 8, 12, 16, and 20 mg phenol L^−1^, respectively (Figure 4B). Subsequently, during the entire incubation period, phenol concentrations of 16 and 20 mg phenol L^−1^ consistently exhibited the effective inhibition of NAG activity. PHO activity was much less responsive to phenol addition (Figure 4C).

For long-term drainage experiments, the BDG, NAG, and PHO activities from low-phenolic soils and the soil samples with low- and high-phenolic contents in all treatments are summarized in Supplementary Table S6 and illustrated graphically compared to the control plots in Figures 4D,E. In the control plots, the BDG, NAG, and PHO activities were 34.9, 33.9, and 210.5 μmol MUF released g^−1^ min^−1^, respectively. In the drained area, the concentrations of phenolic compounds at the two sampling sites were 2.8 and 13.8 mg phenol L^−1^. BAG, NAG, and PHO activities were significantly elevated relative to the control plots in the drained plot with low phenolic content (Figure 4D). The activities of BAG, NAG, and PHO were 1.2, 1.6, and 0.6 times those in the control plots, respectively. In the drained area with high phenolic content, the activities of BAG and NAG did not increase due to drainage; instead, a slight decrease in these activities was observed (Figure 4E). However, the PHO activity was still 0.6 times higher than that in the control due to drainage.

## 4 Discussion

### 4.1 Distinctive drought responses in wetland ecosystems

This analysis demonstrated that drought significantly enhanced the activity of P-related enzymes, while not affecting other soil EEAs in wetlands (Figure 2). Intriguingly, this responsive pattern was found to be exclusive to wetland ecosystems, as evidenced by a comparison with recent systematic reviews and meta-analyses (Supplementary Table S5). These comprehensive studies almost consistently demonstrated that the activities of C-, N-, and P-related enzymes decreased differently in response drought in water-unsaturated ecosystems, namely forests, grasslands, and shrublands (Ren et al., 2017; Xiao et al., 2018; Gao et al., 2020; Sun et al., 2020; Deng et al., 2021; Margalef et al., 2021). Among these previous studies, only Ren et al. (2017) synthesized the response of oxidase activity to altered precipitation. Unsurprisingly, decreased precipitation also led to a significant decline of oxidase activity by 11% in global forests. The initial soil moisture levels may determine the response pattern of soil EEAs to water scarcity and aeration during subsequent drought events (Henry, 2012; Xiao et al., 2018; Zuccarini et al., 2023), likely resulting in significantly different effects of drought on soil EEAs between water-unsaturated ecosystems, such as forests and grasslands, and waterlogged ecosystems, such as wetlands.

In most cases, drought in water-unsaturated ecosystems often leads to a reduction in soil EEAs (Sardans and Peñuelas, 2005). This reduction is primarily because soil water scarcity hampers the diffusion of enzymes and substrates (Margalef et al., 2021; Zuccarini et al., 2023). The release and diffusion of extracellular enzymes from their parent cells rely on the occurrence of Brownian motion in free solutions (Burns et al., 2013). Column transport experiments conducted by Guber et al. (2022) demonstrated that hydrolytic enzymes can be transported through soil pores by water fluxes. However, during drought, the water in many soil pores is replaced with air, disrupting the water pathways and limiting free enzyme motion (Or et al., 2007). Moreover, the drying of the soil increases the presence of air-filled pores and enhances the tortuosity of water films on solid particles, thereby reducing substrate diffusivity (Moldrup et al., 2001). These dual constraints on enzyme and substrate motility under dry conditions lead to a reduction in soil EEAs. In addition to diffusion changes of enzymes and substrates, severe drought can also strongly limit enzymatic production by changing the metabolic strategy, abundance, and diversity of microorganisms and plants (Santonja et al., 2017; Margalef et al., 2021). In hardwood forests, Baldrian et al. (2010) observed a seasonal decline in soil enzyme activity and microbial abundance in responses to soil water loss. Similarly, a drought experiment conducted in grassland showed that soil water content was the most important factor explaining the decline in soil EEAs and changes in bacterial community composition (Gao et al., 2021). Severe drought also strongly affects plant metabolism, inducing roots to prioritize investment in water retention strategies, ultimately leading to a reduction in the release of extracellular enzyme release (Margalef et al., 2021).

In waterlogged ecosystems, the presence of oxygen constraints associated with waterlogging substantially restricts enzymatic decomposition, considering that many enzyme-producing strains and enzymatic reactions require aerobic conditions (Fenner and Freeman, 2011). To underscore the inhibition of anoxia on wetland EEAs, this study briefly compared the wetland enzyme dataset with the average global enzyme activity as reported by Sinsabaugh et al. (2008). The results showed that, under pristine conditions, the activities of C-, N-, and P-related enzymes, as well as oxidase, in the wetland were 1.8–39.3, 2.4–48.3, 1.03–11.7, and 2.3–2966.6 times lower than the average values of global terrestrial ecosystems, respectively. Predictably, drought-induced water table drawdown allows the penetration of oxygen into previously anoxic sites, thereby increasing its availability to soil enzymes and microorganisms (Fenner and Freeman, 2011; Henry, 2012). This relief from oxygen restriction promotes the production and activity of soil EEAs. Moreover, drought can increase the soil redox potential, which has been shown to be significantly related to the increase of EEAs under drying conditions (Vo and Kang, 2013).

However, the results indicate that not all EEAs undergo changes to adapt to increased oxygen exposure during drought in global wetland ecosystems. These results contrast our first hypothesis but supports our second hypothesis. This suggests that other competing mechanisms may govern soil EEAs during drought, alongside the relief of oxygen restriction.

### 4.2 Regulation of phenolic compounds

Phenolic compounds are a class of organic compounds characterized by the presence of aromatic rings directly bonded to one or more hydroxyl functional groups (Dunn and Freeman, 2017). These compounds primarily originate from the decomposition of plant residues and the release of root exudates (Hättenschwiler and Vitousek, 2000). Phenolic compounds can exert both direct and indirect effects on soil EEAs through various non-mutually exclusive mechanisms.

Directly, phenolic compounds can inactivate enzymes via protein precipitation and competitive inhibition (Freeman et al., 2001; Fenner and Freeman, 2020). Indirectly, they have been found to alter the composition and activity of microorganisms, thereby influencing enzyme production and nutrient cycling (Hättenschwiler and Vitousek, 2000). In addition, phenolic compounds can form complexes with soil organic compounds, such as polyphenol–protein complexes, leading to significant impacts on the availability of enzymatic substrates (Hättenschwiler and Vitousek, 2000). Recently, Zhao et al. (2023) have also reported that certain phenolic acids can enhance metal–organic associations and further exacerbate the unavailability of substrates. Consequently, phenolic compounds are generally considered the rate-limiting agents in enzyme-mediated decomposition.

Our results demonstrated that drought has significantly increased the concentration of phenolic compounds as a whole in global wetlands (Figure 2C). This accumulation of polyphenolic compounds could be primarily attributed to the growth of high-phenolic dominant species (Wang et al., 2015), as indicated by their responses in subgroups (Figure 3E). When the dataset was grouped based on dominant plants, drought significantly increased the content of phenolic compounds in wetland-dominated sedges and wood species, while it had no effect on the concentration of phenolic compounds in moss-dominated wetlands. The concentrations of phenolic compounds in moss were considerably lower than in the leachates of vascular plants (Wang et al., 2015). In wet soil, water-table drawdown favors the seed germination, seedling establishment, and root respiration of vascular plants, resulting in a higher input of high-phenolic litter (Wang et al., 2015; Zacks et al., 2018). However, changes in plant growth or even a shift in dominant species typically require a prolonged duration of drought (Li et al., 2020), which aligns with our observation that long-term drought has a more pronounced effect on increasing the content of phenolic compounds compared to short-term drought. Overall, phenolic inhibition in wetlands, as a mechanism distinct from oxygen exposure, may regulate soil EEAs during drought. Based on this, we hypothesize that variation in enzyme sensitivity to phenolic compounds result in the unique response patterns of C-, N-, and P-related enzymes to drought in global wetlands (second hypothesis).

To test this hypothesis, we conducted a two-week incubation experiment with varying concentrations of phenol solution supplementation (see Section 2.2 in the Data and Methodology for details). The results revealed that the activities of BDG and NAG were consistently inhibited when the concentration of the added phenol solution exceeded 12 and 16 mg phenol L^−1^, respectively (Figures 4A,B). However, even at 20 mg phenol L^−1^, which is about twice the maximum phenol concentration observed in this sampling site under drought conditions, the addition of phenol solution had no significant effect on the PHO activity (Figure 4C). These results suggest that P-related enzymes can tolerate high levels of phenolic compounds, while C- and N-related enzymes may not. In support of this, Dunn and Freeman (2017) reported that the addition of calcium lignosulphonic acid (a high-molecular-weight polyphenol) at an extremely high concentration of 50 g L^−1^ resulted in only a 52% decrease in PHO activity, whereas BDG activity was significantly inhibited, by 78%. However, there is not much literature on this topic. To the best of our knowledge, our results represent the first demonstration of varying degree of sensitivity exhibited by different hydrolytic enzymes to phenolic compounds.

To further investigate the trade-off of soil EEAs between their promotion due to oxygen exposure and their inhibition caused by phenolic compounds during drought, we compared the activity of key hydrolytic enzymes in a long-term drainage peatland while considering varying soil phenolic concentrations (Figures 4D,E). The peatland area had undergone 35 years of drainage, resulting in the replacement of dominant *Sphagnum* species with clusters of shrubs (Li et al., 2020). The concentration of phenolic compounds in the peat beneath shrubs was significantly higher compared to that obtained from *Sphagnum* hummocks. In plots with low phenolic concentration, water-table drawdown significantly promoted the activities of BAG, NAG, and PHO. Conversely, in plots with a high-phenolic concentration, PHO activity increased by 60%, while the activities of other enzymes did not change significantly even after a prolonged duration of drainage. These results further confirmed that the accumulation of phenolic compounds due to the shift in vegetation composition seems to exert control over certain soil EEAs during drought (Wang et al., 2015; Fenner and Freeman, 2020).

**Figure 5:**
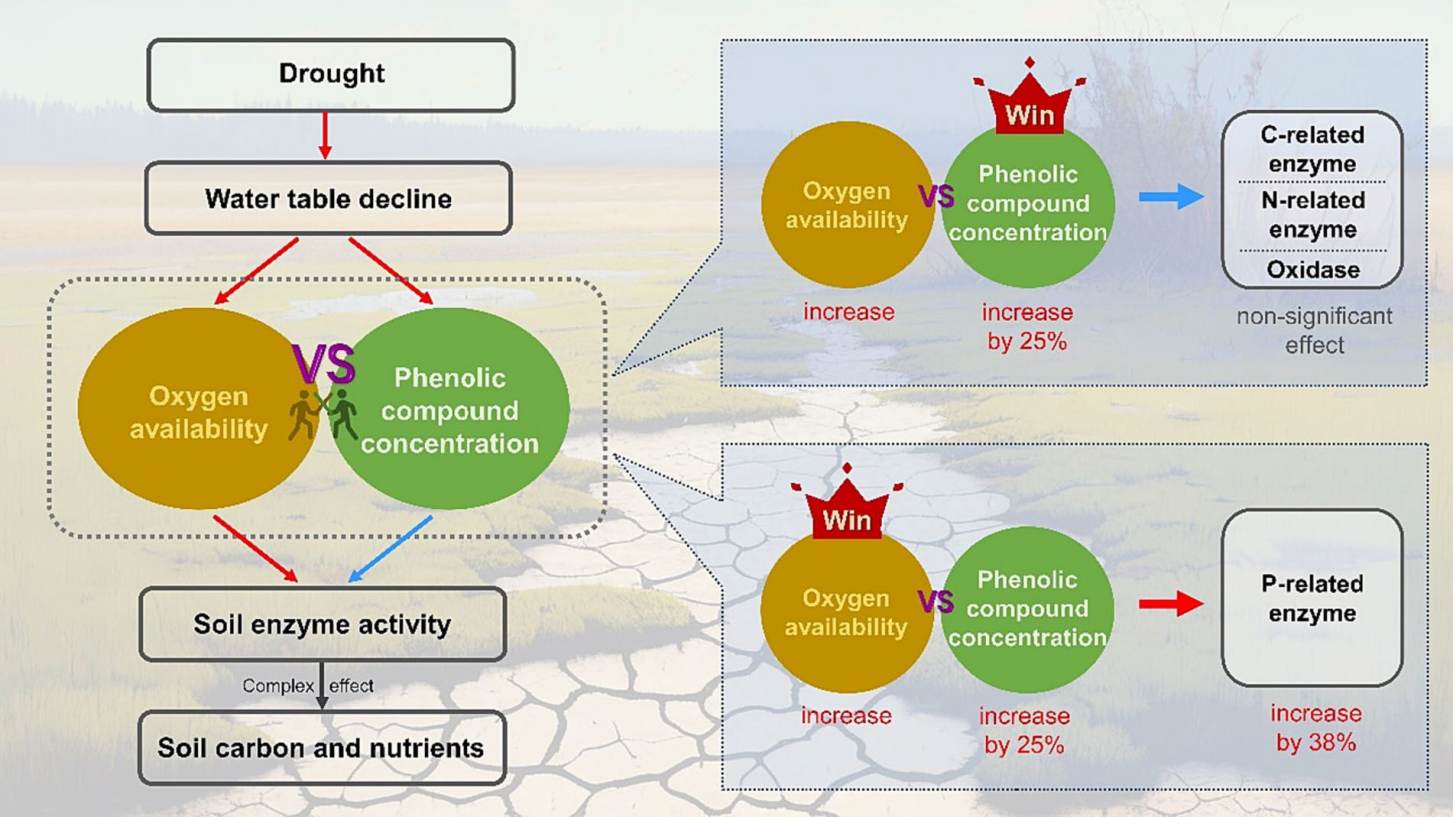
Conceptual model showing the effects of oxygen availability and phenolic compounds concentration on activities of the soil extracellular enzymes. Red and blue arrows indicate positive and negative influences, respectively.

In summary, for wetland ecosystems, the unsaturated conditions caused by drought facilitate the penetration of oxygen into the soil, which can promote the production and activities of soil EEAs (Henry, 2012; Zuccarini et al., 2023). However, drought can also enhance the accumulation of phenolic compounds by changing plant growth or even spurring vegetation succession, which may inhibit the activities of decomposition-related enzymes through various mechanisms (Wang et al., 2015; Fenner and Freeman, 2020). These two competing mechanisms may have unforeseen effects on soil EEAs (Figure 5). Furthermore, due to the high resistance of certain enzymes to phenolic compounds, their activities could be promoted by oxygen flux, such as P-related enzymes, while most extracellular enzymes may not retain their activities, including C- and N-related enzymes. As a result, enzyme-mediated decomposition exhibits a degree of resilience, allowing the concentration of soil carbon and nutrients to remain relatively stable during drought in wetland ecosystems (Figure 3).

### 4.3 Research limitations and future works

In the present study, we conclude that wetlands seem to be complex adaptive systems with great resilience to cope with drought stress. However, it is important to acknowledge the following limitations that may impact the results of the present analyses. Therefore, we endeavor to offer potential trends and directions for future research. First, in this study, phenol was used as a representative compound for polyphenols in incubation experiments, aiming to assess the varying sensitivity of EEAs to phenolic compounds. There are about 8,000 known phenolic structures found in plants, and it is not uncommon to detect over 20 phenolic compounds in soils (Hättenschwiler and Vitousek, 2000; Dai and Mumper, 2010). For example, phenolic acids such as gallic, caffeic, and ferulic acids, tend to accumulate in the root zone and have been found to indirectly suppress the soil EEAs by inhibiting microbial metabolism (Zhao et al., 2021). As protein-binding phenolic compounds, tannins (medium-molecular-weight phenolic compounds with a molecular weight range of 500–3,000) lower nutrient mobilization via proteotoxic effects on enzymes (Hättenschwiler and Vitousek, 2000). Furthermore, Dunn and Freeman (2017) provided compelling evidence that high-molecular-weight phenolic compounds, such as calcium lignosulphonic acid, with a molecular weight of 49,100, exhibited a more pronounced inhibitory effect on soil EEAs and organic matter decomposition. Several uncertainties remain regarding the significance of specific groups of phenolic compounds. Moreover, it remains unknown whether the types of polyphenols that regulate enzyme activity are consistent across different wetland types. In addition, phenolic compounds may have antimicrobial effects, stimulatory effects, or both, being able to simultaneously serve as the source of microbial food and toxicity (Stanek et al., 2021). Thus, the regulation of phenolic compounds on enzyme activity under drought condition may be more intricate and multifaceted than currently acknowledged. An additional limitation of this study is the reliance on a single field site in Northeast China for the drainage experiment. This approach may not adequately capture the variability in soil enzyme responses to phenolic compounds across different wetland ecosystems. Although the long-term drainage experiment conducted in the peatland of Northeast China supports the findings from the meta-analysis, the applicability of these results to other regions requires further investigation.

Second, in the present study, significant increases in the activities of P-related enzymes have been observed in response to drought, and their activities appeared to be less susceptible to the toxicity of phenolic compounds compared to other enzymes. Consequently, the fate of phosphorus in global wetlands under future climatic regimes is concerning, despite the absence of significant changes observed in total phosphorus in this study. Wetlands harbor a substantial reservoir of soil organic phosphorus due to incomplete decomposition of biological residues under waterlogged conditions (Schlesinger and Bernhardt, 2020). The mineralization of organic phosphorus can be facilitated by a series of phosphatases, releasing a substantial amount of bioavailable phosphorus (Bünemann, 2015). The phosphorus nutrient status of decomposers is expected to be altered as a result of the increment in the frequency and intensity of droughts in the future, which brings significant uncertainty into the estimation of wetland carbon sink potential. In addition to being compensated and amplified by climate change, the activities of soil P-related enzymes also depend on the initial soil conditions (Margalef et al., 2021), metallic oxides (Zhao et al., 2021), and plant metabolism (Chen et al., 2022), which may lead to complex and unpredictable response patterns. Consequently, further multi-factor research is necessary to elucidate these interactions. Besides, the organic phosphorus regulated by phosphorus-related enzymes likely constitutes only a small portion of the total soil phosphorus. Consequently, the observed mismatch between phosphorus-related enzymes and total phosphorus is not unexpected.

Additionally, in our study we did not observe significant responses of oxidases to drought in global wetlands, which is a surprising result given that oxidation catalysis typically relies on the availability of oxygen (Freeman et al., 2004). Despite conducting a relatively comprehensive review of the current literature, no substantial explanation was identified. Oxidases are recognized for their involvement in carbon cycling by oxidizing the recalcitrant fractions of soil organic matter (Li et al., 2020). In particular, certain oxidases, such as phenol oxidase, play an important role in regulating the biological toxicity of polyphenols (Freeman et al., 2001). The current knowledge regarding the biological properties, catalytic conditions, and dynamics of oxidases under aerobic conditions is severely insufficient.

Lastly, although phenolic compounds exert a powerful inhibitory effect on soil EEAs under drought conditions, our stance remains in support of the notion that prolonged and severe drought poses a significant risk to the conservation of organic matter in wetlands. This is because plants, as the primary source of soil phenolic compounds, ultimately succumb to mortality in response to severe water deficits (Mantova et al., 2022). The response of soil EEAs to drought may therefore be nonlinear, and there may be a critical tipping point for EEAs (Scheffer and Carpenter, 2003; Jassey et al., 2018). An enzyme may remain stable before reaching the tipping point, as phenolic compounds inhibit its activity. However, once this threshold is surpassed, a significant alteration occurs in both enzyme activity and nutrient cycling due to ecological function breakdown, which might be difficult to reverse. The precise location of this tipping point remains unclear, hindering the assessment of the risks and benefits associated with drought in wetland ecosystems.

## Data availability statement

The raw data supporting the conclusions of this article will be made available by the authors, without undue reservation.

## Author contributions

TL: Writing – original draft, Writing – review & editing, Conceptualization, Formal analysis, Funding acquisition, Investigation, Methodology. LG: Writing – review & editing, Data curation, Formal analysis, Investigation, Methodology, Validation. RZ: Writing – review & editing, Data curation, Validation. CP: Writing – review & editing. XZ: Writing – review & editing, Conceptualization. PL: Writing – review & editing, Formal analysis. ZL: Writing – review & editing. HS: Writing – review & editing, Data curation. JT: Writing – review & editing. CZ: Writing – review & editing. QL: Writing – review & editing, Formal analysis, Investigation. MW: Writing – review & editing, Conceptualization, Funding acquisition. ZZ: Writing – review & editing, Writing – review & editing, Funding acquisition.

## Footnotes

^1^http://apps.webofknowledge.com

^2^http://www.cnki.net

## References

[ref1] Angert A. Biraud S. Bonfils C. Henning C. C. Buermann W. Pinzon J.. (2005). Drier summers cancel out the CO_2_ uptake enhancement induced by warmer springs. PNAS Nexus 102, 10823–10827. doi: 10.1073/pnas.0501647102, PMID: 16043702 PMC1180508

[ref2] Baldrian P. Merhautová V. Petránková M. Cajthaml T. Šnajdr J. (2010). Distribution of microbial biomass and activity of extracellular enzymes in a hardwood forest soil reflect soil moisture content. Appl. Soil Ecol. 46, 177–182. doi: 10.1016/j.apsoil.2010.08.013

[ref3] Berdugo M. Delgado-Baquerizo M. Soliveres S. Hernandez-Clemente R. Zhao Y. C. Gaitan J. J.. (2020). Global ecosystem thresholds driven by aridity. Science 367, 787–790. doi: 10.1126/science.aay5958, PMID: 32054762

[ref4] Box J. D. (1983). Investigation of the Folin-Ciocalteau phenol reagent for the determination of polyphenolic substances in natural waters. Water Res. 17, 511–525. doi: 10.1016/0043-1354(83)90111-2

[ref5] Brockett B. F. T. Prescott C. E. Grayston S. J. (2012). Soil moisture is the major factor influencing microbial community structure and enzyme activities across seven biogeoclimatic zones in western Canada. Soil Biol. Biochem. 44, 9–20. doi: 10.1016/j.soilbio.2011.09.003

[ref6] Bu Z. J. Rydin H. Chen X. (2011). Direct and interaction-mediated effects of environmental changes on peatland bryophytes. Oecologia 166, 555–563. doi: 10.1007/s00442-010-1880-1, PMID: 21170747

[ref7] Bu Z. J. Sundberg S. Feng L. Li H. K. Zhao H. Y. Li H. C. (2017). The methuselah of plant diaspores: spores can survive in nature for centuries. New Phytol. 214, 1398–1402. doi: 10.1111/nph.14575, PMID: 28401984

[ref8] Bünemann E. K. (2015). Assessment of gross and net mineralization rates of soil organic phosphorus – a review. Soil Biol. Biochem. 89, 82–98. doi: 10.1016/j.soilbio.2015.06.026

[ref9] Burns R. G. DeForest J. L. Marxsen J. Sinsabaugh R. L. Stromberger M. E. Wallenstein M. D.. (2013). Soil enzymes in a changing environment: current knowledge and future directions. Soil Biol. Biochem. 58, 216–234. doi: 10.1016/j.soilbio.2012.11.009

[ref10] Chen X. Chen H. Y. H. Chang S. X. (2022). Meta-analysis shows that plant mixtures increase soil phosphorus availability and plant productivity in diverse ecosystems. Nat. Ecol. Evol. 6, 1112–1121. doi: 10.1038/s41559-022-01794-z, PMID: 35760890

[ref11] Choat B. Brodribb T. J. Brodersen C. R. Duursma R. A. López R. Medlyn B. E. (2018). Triggers of tree mortality under drought. Nature 558, 531–539. doi: 10.1038/s41586-018-0240-x, PMID: 29950621

[ref12] Cui Y. X. Moorhead D. L. Guo X. B. Peng S. S. Wang Y. Q. Zhang X. C.. (2021). Stoichiometric models of microbial metabolic limitation in soil systems. Glob. Ecol. Biogeogr. 30, 2297–2311. doi: 10.1111/geb.13378

[ref13] Dai J. Mumper R. J. (2010). Plant phenolics: extraction, analysis and their antioxidant and anticancer properties. Molecules 15, 7313–7352. doi: 10.3390/molecules15107313, PMID: 20966876 PMC6259146

[ref14] Deng L. Peng C. H. Kim D. G. Li J. W. Liu Y. L. Hai X. Y.. (2021). Drought effects on soil carbon and nitrogen dynamics in global natural ecosystems. Earth-Sci. Rev. 214:103501. doi: 10.1016/j.earscirev.2020.103501

[ref15] Doughty C. E. Metcalfe D. B. Girardin C. A. J. Amézquita F. F. Cabrera D. G. Huasco W. H.. (2015). Drought impact on forest carbon dynamics and fluxes in Amazonia. Nature 519, 78–82. doi: 10.1038/nature14213, PMID: 25739631

[ref16] Dunn C. Freeman C. (2017). The role of molecular weight in the enzyme-inhibiting effect of phenolics: the significance in peatland carbon sequestration. Ecol. Eng. 114, 162–166. doi: 10.1016/j.ecoleng.2017.06.036

[ref17] Dunn C. Jones T. G. Girard A. Freeman C. (2014). Methodologies for extracellular enzyme assays from wetland soils. Wetlands 34, 9–17. doi: 10.1007/s13157-013-0475-0

[ref18] Fenner N. Freeman C. (2011). Drought-induced carbon loss in peatlands. Nat. Geosci. 4, 895–900. doi: 10.1038/Ngeo1323

[ref19] Fenner N. Freeman C. (2020). Woody litter protects peat carbon stocks during drought. Nat. Clim. Chang. 10, 363–369. doi: 10.1038/s41558-020-0727-y

[ref20] Freeman C. Ostle N. J. Fenner N. Kang H. (2004). A regulatory role for phenol oxidase during decomposition in peatlands. Soil Biol. Biochem. 36, 1663–1667. doi: 10.1016/j.soilbio.2004.07.012

[ref21] Freeman C. Ostle N. Kang H. (2001). An enzymic 'latch' on a global carbon store. Nature 409:149. doi: 10.1038/35051650, PMID: 11196627

[ref22] Gao D. C. Bai E. Li M. H. Zhao C. H. Yu K. L. Hagedorn F. (2020). Responses of soil nitrogen and phosphorus cycling to drying and rewetting cycles: a meta-analysis. Soil Biol. Biochem. 148:107896. doi: 10.1016/j.soilbio.2020.107896

[ref23] Gao W. L. Reed S. C. Munson S. M. Rui Y. C. Fan W. Y. Zhang Z. Z.. (2021). Responses of soil extracellular enzyme activities and bacterial community composition to seasonal stages of drought in a semiarid grassland. Geoderma 401:115327. doi: 10.1016/j.geoderma.2021.115327

[ref24] Guber A. Blagodatskaya E. Kravchenko A. (2022). Are enzymes transported in soils by water fluxes? Soil Biol. Biochem. 168:108633. doi: 10.1016/j.soilbio.2022.108633

[ref25] Guo X. Y. Peng C. H. Li T. Huang J. J. Song H. X. Zhu Q. A.. (2021). The effects of drought and re-watering on non-structural carbohydrates of *Pinus tabulaeformis* seedlings. Biology-Basel 10:281. doi: 10.3390/biology10040281, PMID: 33808347 PMC8066268

[ref26] Hättenschwiler S. Vitousek P. M. (2000). The role of polyphenols in terrestrial ecosystem nutrient cycling. Trends Ecol. Evol. 15, 238–243. doi: 10.1016/s0169-5347(00)01861-9, PMID: 10802549

[ref27] Haugwitz M. S. Michelsen A. Priemé A. (2016). The legacy of climate change effects: previous drought increases short-term litter decomposition rates in a temperate mixed grass- and shrubland. Plant Soil 408, 183–193. doi: 10.1007/s11104-016-2913-2

[ref28] Hedges L. V. Gurevitch J. Curtis P. S. (1999). The meta-analysis of response ratios in experimental ecology. Ecology 80, 1150–1156. doi: 10.1890/0012-9658(1999)080[1150,Tmaorr]2.0.Co;2

[ref29] Henry H. A. L. (2012). Soil extracellular enzyme dynamics in a changing climate. Soil Biol. Biochem. 47, 53–59. doi: 10.1016/j.soilbio.2011.12.026

[ref30] Jassey V. E. J. Reczuga M. K. Zielinska M. Slowinska S. Robroek B. J. M. Mariotte P.. (2018). Tipping point in plant-fungal interactions under severe drought causes abrupt rise in peatland ecosystem respiration. Glob. Chang. Biol. 24, 972–986. doi: 10.1111/gcb.13928, PMID: 28991408

[ref31] Laiho R. Vasander H. Penttilä T. Laine J. (2003). Dynamics of plant-mediated organic matter and nutrient cycling following water-level drawdown in boreal peatlands. Glob. Biogeochem. Cycle 17:1053. doi: 10.1029/2002gb002015

[ref32] Li T. Ge L. M. Huang J. J. Yuan X. Peng C. H. Wang S. Z.. (2020). Contrasting responses of soil exoenzymatic interactions and the dissociated carbon transformation to short- and long-term drainage in a minerotrophic peatland. Geoderma 377:114585. doi: 10.1016/j.geoderma.2020.114585

[ref33] Liu D. J. Ogaya R. Barbeta A. Yang X. H. Penuelas J. (2015). Contrasting impacts of continuous moderate drought and episodic severe droughts on the aboveground-biomass increment and litterfall of three coexisting Mediterranean woody species. Glob. Chang. Biol. 21, 4196–4209. doi: 10.1111/gcb.13029, PMID: 26149833

[ref34] Mantova M. Herbette S. Cochard H. Torres-Ruiz J. M. (2022). Hydraulic failure and tree mortality: from correlation to causation. Trends Plant Sci. 27, 335–345. doi: 10.1016/j.tplants.2021.10.003, PMID: 34772610

[ref35] Margalef O. Sardans J. Maspons J. Molowny-Horas R. Fernández-Martínez M. Janssens I. A.. (2021). The effect of global change on soil phosphatase activity. Glob. Chang. Biol. 27, 5989–6003. doi: 10.1111/gcb.15832 34383341

[ref36] Moldrup P. Olesen T. Komatsu T. Schjonning P. Rolston D. E. (2001). Tortuosity, diffusivity, and permeability in the soil liquid and gaseous phases. Soil Sci. Soc. Am. J. 65, 613–623. doi: 10.2136/sssaj2001.653613x

[ref37] Moorhead D. L. Sinsabaugh R. L. (2006). A theoretical model of litter decay and microbial interaction. Ecol. Monogr. 76, 151–174. doi: 10.1890/0012-9615(2006)076[0151:Atmold]2.0.Co;2

[ref38] Muller L. M. Bahn M. (2022). Drought legacies and ecosystem responses to subsequent drought. Glob. Chang. Biol. 28, 5086–5103. doi: 10.1111/gcb.16270, PMID: 35607942 PMC9542112

[ref39] Or D. Smets B. F. Wraith J. M. Dechesne A. Friedman S. P. (2007). Physical constraints affecting bacterial habitats and activity in unsaturated porous media – a review. Adv. Water Resour. 30, 1505–1527. doi: 10.1016/j.advwatres.2006.05.025

[ref40] Ren C. J. Zhao F. Z. Shi Z. Chen J. Han X. H. Yang G. H.. (2017). Differential responses of soil microbial biomass and carbon-degrading enzyme activities to altered precipitation. Soil Biol. Biochem. 115, 1–10. doi: 10.1016/j.soilbio.2017.08.002

[ref41] Santonja M. Fernandez C. Proffit M. Gers C. Gauquelin T. Reiter I. M.. (2017). Plant litter mixture partly mitigates the negative effects of extended drought on soil biota and litter decomposition in a Mediterranean oak forest. J. Ecol. 105, 801–815. doi: 10.1111/1365-2745.12711

[ref42] Sardans J. Peñuelas J. (2005). Drought decreases soil enzyme activity in a Mediterranean *Quercus ilex* L. forest. Soil Biol. Biochem. 37, 455–461. doi: 10.1016/j.soilbio.2004.08.004

[ref43] Scheffer M. Carpenter S. R. (2003). Catastrophic regime shifts in ecosystems: linking theory to observation. Trends Ecol. Evol. 18, 648–656. doi: 10.1016/j.tree.2003.09.002

[ref44] Schlesinger W. H. Bernhardt E. S. (2020). “Chapter 7 - wetland ecosystems” in Biogeochemistry: An analysis of global change. 4th ed (London, UK: Academic Press)

[ref45] Sinsabaugh R. L. Lauber C. L. Weintraub M. N. Ahmed B. Allison S. D. Crenshaw C.. (2008). Stoichiometry of soil enzyme activity at global scale. Ecol. Lett. 11, 1252–1264. doi: 10.1111/j.1461-0248.2008.01245.x, PMID: 18823393

[ref46] Stanek M. Zubek S. Stefanowicz A. M. (2021). Differences in phenolics produced by invasive *Quercus rubra* and native plant communities induced changes in soil microbial properties and enzymatic activity. For. Ecol. Manag. 482:118901. doi: 10.1016/j.foreco.2020.118901

[ref47] Sun Y. Liao J. H. Zou X. M. Xu X. A. Yang J. Y. Chen H. Y. H.. (2020). Coherent responses of terrestrial C:N stoichiometry to drought across plants, soil, and microorganisms in forests and grasslands. Agric. For. Meteorol. 292:108104. doi: 10.1016/j.agrformet.2020.108104

[ref48] Talbot J. Richard P. J. H. Roulet N. T. Booth R. K. (2010). Assessing long-term hydrological and ecological responses to drainage in a raised bog using paleoecology and a hydrosequence. J. Veg. Sci. 21, 143–156. doi: 10.1111/j.1654-1103.2009.01128.x

[ref49] Tsiafouli M. A. Monokrousos N. Sgardelis S. P. (2018). Drought in spring increases microbial carbon loss through respiration in a Mediterranean pine forest. Soil Biol. Biochem. 119, 59–62. doi: 10.1016/j.soilbio.2018.01.010

[ref50] Tuck S. L. Winqvist C. Mota F. Ahnström J. Turnbull L. A. Bengtsson J. (2014). Land-use intensity and the effects of organic farming on biodiversity: a hierarchical meta-analysis. J. Appl. Ecol. 51, 746–755. doi: 10.1111/1365-2664.12219, PMID: 25653457 PMC4299503

[ref51] Viechtbauer W. (2010). Conducting meta-analyses in R with the metafor package. J. Stat. Softw. 36, 1–48. doi: 10.18637/jss.v036.i03

[ref52] Vo N. X. Q. Kang H. (2013). Regulation of soil enzyme activities in constructed wetlands under a short-term drying period. Chem. Ecol. 29, 146–165. doi: 10.1080/02757540.2012.711323

[ref53] Wan X. Xiang W. Wan N. Yan S. Bao Z. Y. Wang Y. L. (2018). Complexation and reduction of iron by phenolic substances: implications for transport of dissolved Fe from peatlands to aquatic ecosystems and global iron cycling. Chem. Geol. 498, 128–138. doi: 10.1016/j.chemgeo.2018.09.019

[ref54] Wang H. J. Richardson C. J. Ho M. C. (2015). Dual controls on carbon loss during drought in peatlands. Nat. Clim. Chang. 5, 584–587. doi: 10.1038/nclimate2643

[ref55] Xiao W. Chen X. Jing X. Zhu B. A. (2018). A meta-analysis of soil extracellular enzyme activities in response to global change. Soil Biol. Biochem. 123, 21–32. doi: 10.1016/j.soilbio.2018.05.001

[ref56] Zacks G. Greet J. Walsh C. J. Raulings E. (2018). The flooding tolerance of two critical habitat-forming wetland shrubs, *Leptospermum lanigerum* and *Melaleuca squarrosa*, at different life history stages. Aust. J. Bot. 66, 500–510. doi: 10.1071/Bt18039

[ref57] Zhang J. W. Feng Y. Z. Maestre F. T. Berdugo M. Wang J. T. Coleine C.. (2023). Water availability creates global thresholds in multidimensional soil biodiversity and functions. Nat. Ecol. Evol. 7, 1002–1011. doi: 10.1038/s41559-023-02071-3, PMID: 37169879

[ref58] Zhao Y. P. Liu C. Z. Li X. Q. Ma L. X. Zhai G. Q. Feng X. J. (2023). *Sphagnum* increases soil's sequestration capacity of mineral-associated organic carbon via activating metal oxides. Nat. Commun. 14:5052. doi: 10.1038/s41467-023-40863-0, PMID: 37598219 PMC10439956

[ref59] Zhao Y. P. Liu C. Z. Wang S. M. Wang Y. Y. Liu X. Q. Luo W. Q.. (2021). “Triple locks” on soil organic carbon exerted by sphagnum acid in wetlands. Geochim. Cosmochim. Acta 315, 24–37. doi: 10.1016/j.gca.2021.09.028

[ref60] Zuccarini P. Sardans J. Asensio L. Penuelas J. (2023). Altered activities of extracellular soil enzymes by the interacting global environmental changes. Glob. Chang. Biol. 29, 2067–2091. doi: 10.1111/gcb.16604, PMID: 36655298

