# Supplementary material for: Phenolic compounds weaken the impact of drought on soil enzyme activity in global wetlands
Source: Front Microbiol. 2024 Mar 8;15:1372866. doi: 10.3389/fmicb.2024.1372866 (PMC10957752; doi:10.3389/fmicb.2024.1372866)
Supplement: Supplementary file 2 [file Table_2.DOCX]

Supplementary Materials

**Phenolic compounds weaken the impact of drought on soil enzyme activity in global wetlands**

**Supplementary Figure S1 PRISMA flow chart showing the procedure of selecting studies.**


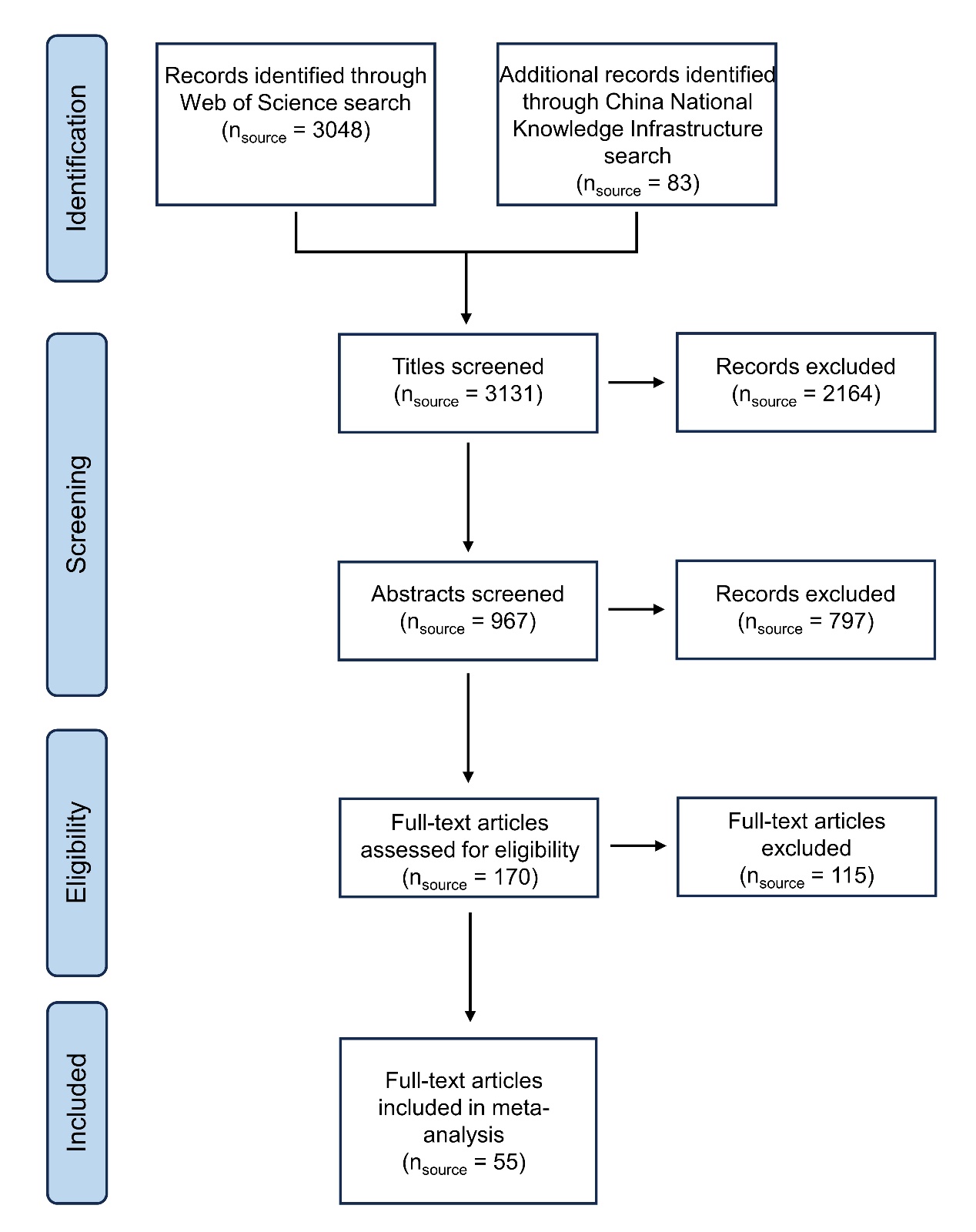


**Supplementary Table S1** Search strings used to find relevant publications for the meta-analysis.

| Topic item | Search string |
| --- | --- |
| TS1 | “wetland*” OR “peatland*” OR “fen*” OR “bog*” OR “swamp*” OR “marsh*” OR “mire*” |
| TS2 | “drought” OR “drainage” OR “dry*” OR “water tress” OR “water treatment” OR “altered precipitation” OR “decreased precipitation” OR “rainout shelter” OR “rainfall reduction” OR “decreased rainfall” |
| TS3 | “enzyme* activit*” OR “enzymatic activit*” OR “hydrolase* activit*” OR “hydrolytic ferment* activit*” OR “hydrolys* activit*” OR “oxidase* activit*” OR “peroxidase* activit*” OR “catalase* activit*” OR “laccase* activit*” OR “exoenzyme* activit*” OR “ectoenzyme* activit*” OR “micro* activit*” OR “enzyme* respon*” OR “enzymatic respon*” OR “hydrolase* respon*” OR “hydrolytic ferment* respon*” OR “hydrolys* respon*” OR “oxidase* respon*” OR “oxidase* respon*” OR “peroxidase* respon*” OR “catalase* respon*” OR “laccase* respon*” OR “exoenzyme* respon*” OR “ectoenzyme* respon*” OR “micro* respon*” OR “enzyme* active” OR “enzymatic active” OR “hydrolase* active” OR “hydrolytic ferment* active” OR “hydrolys* active” OR “oxidase* active” OR “oxidase* active*” OR “peroxidase* active*” OR “catalase* active*” OR “laccase* active*” OR “exoenzyme* active” OR “ectoenzyme* active” OR “micro* active” OR “enzyme* expression*” OR “enzymatic expression*” OR “hydrolase* expression*” OR “hydrolytic ferment* expression*” OR “oxidase* expression*” OR “oxidase* expression*” OR “peroxidase* expression*” OR “catalase* expression*” OR “laccase* expression*” OR “hydrolys* expression*” OR “exoenzyme* expression*” OR “ectoenzyme* expression*” OR “micro* expression*” |
| TS4 | “phenolic*” OR “tannin*” OR “flavonoid” OR “phenolic* acid*” OR “quercetin*” OR “kaempferol*” OR “anthocyanins*” OR “lignin*” OR “catechin*” OR “polyphenolic*” OR “rutin*” OR “chlorogenic* acid*” OR “polyphenol*” OR “caffeic* acid*” OR “benzoic* acid*” OR “vanillic* acid*” OR “dihydroxybenzoic* acid*” OR “ferulic* acid*” OR “salicylic* acid*” OR “coumaric* acid*” OR “syringate*” |

**Supplementary Table S2** Total heterogeneity in weighted effect sizes of activities of soil enzymes, concentration of phenolic compounds, and soil properties.

| Observation | *Q_t_* | *P*-value |
| --- | --- | --- |
| *Soil extracellular enzyme* |  |  |
| Hydrolytic enzyme | 71823.3 | < 0.001 |
| C-related enzyme | 9210.4 | < 0.001 |
| N-related enzyme | 12582.4 | < 0.001 |
| P-related enzyme | 49686.9 | < 0.001 |
| Oxidase | 3709.3 | < 0.001 |
| *Phenolics* |  |  |
| Phenolic compound | 962.3 | < 0.001 |
| *Soil properties* |  |  |
| Total carbon | 423.7 | < 0.001 |
| Total organic carbon | 7562.0 | < 0.001 |
| Dissolved organic carbon | 775.2 | < 0.001 |
| Total nitrogen | 4471.6 | < 0.001 |
| Total phosphorus | 3530.3 | < 0.001 |
| pH | 2459.3 | < 0.001 |
| Microbial biomass carbon | 1491.3 | < 0.001 |

**Supplementary Table S3** Results for publication bias. N is the number of studies for a group. A significant correlation indicates possible publication bias.

| Observation | N | *P*-value |
| --- | --- | --- |
| *Soil extracellular enzyme* |  |  |
| C-related enzyme | 35 | 0.62 |
| N-related enzyme | 22 | 0.27 |
| P-related enzyme | 26 | 0.79 |
| Oxidase | 28 | 0.42 |
| *Phenolics* |  |  |
| Phenolic compound | 11 | 0.09 |
| *Soil properties* |  |  |
| Total carbon | 8 | 0.95 |
| Total organic carbon | 14 | 0.89 |
| Dissolved organic carbon | 13 | 0.42 |
| Total nitrogen | 17 | 0.67 |
| Total phosphorus | 14 | 0.38 |
| pH | 22 | 0.56 |
| Microbial biomass carbon | 9 | 0.40 |

**Supplementary Table S4** Changes in the activities of soil extracellular enzymes.

| Observation | Sample size | Weighted mean effect size | Lower confidence interval | Upper confidence interval |
| --- | --- | --- | --- | --- |
| β-1,4-glucosidase | 91 | -0.02 | -0.37 | 0.32 |
| α-1,4-glucosidase | 10 | 0.11 | 0.03 | 0.19 |
| β-D-cellobiohydrolase | 15 | -0.35 | -1.02 | 0.35 |
| β-1,4-xylosidase | 13 | 0.09 | -0.29 | 0.48 |
| β-1,4-N-acetylglucosaminidase | 49 | -0.12 | -0.62 | 0.37 |
| Phenol oxidase | 105 | -0.03 | -0.30 | 0.24 |
| Peroxidase | 22 | -0.01 | -0.27 | 0.25 |

**Supplementary Table S5** Responses of soil extracellular enzyme activities in forests, grasslands, and shrublands.

| Reference | Ecosystem | C-related enzyme | N-related enzyme | P-related enzyme | Hydrolytic enzyme | Oxidase |
| --- | --- | --- | --- | --- | --- | --- |
| Deng et al., 2021 | Forest | Non-significant decrease | Significant decrease | NA | NA | NA |
|  | Grassland | Non-significant increase | Significant increase | NA | NA | NA |
|  | Shrubland | Non-significant decrease | Non-significant decrease | NA | NA | NA |
| Gao et al., 2020 | Forest | NA | Significant decrease | Significant decrease | NA | NA |
| Margalef et al., 2021 | Multiple ecosystems | NA | NA | Significant decrease | NA | NA |
| Ren et al., 2017 | Forest | NA | NA | NA | Significant increase | Significant decrease |
|  | Grassland | NA | NA | NA | Significant increase | NA |
|  | Shrubland | NA | NA | NA | Non-significant increase | NA |
| Sun et al., 2020 | Forest | Nonsignificant decrease | Non-significant increase | NA | NA | NA |
|  | Grassland | Non-significant decrease | Non-significant decrease | NA | NA | NA |
| Xiao et al., 2018 | Forest | Non-significant increase | Non-significant increase | Non-significant decrease | NA | NA |

*NA represent unclear information.*

**Supplementary Table S6** Activities of β-D-glucosidase, N-acetyl-β-glucosaminidase, and phosphatase after 35-year drainage in the field experiment.

| Enzyme | Treatment | Activity (μmol MUF released g^-1^ min^-1^) | SD |
| --- | --- | --- | --- |
| β-D-glucosidase | control | 23.2 | 1.3 |
|  | drainage + low phenolic content | 21.5* | 1.7 |
|  | drainage + high phenolic content | 50.9 | 3.9 |
| N-acetyl-β-glucosaminidase | control | 23.0 | 1.7 |
|  | drainage + low phenolic content | 22.3* | 3.3 |
|  | drainage + high phenolic content | 59.9 | 4.6 |
| phosphatase | control | 167.5 | 36.6 |
|  | drainage + low phenolic content | 263.4* | 57.6 |
|  | drainage + high phenolic content | 208.6* | 40.9 |

*Asterisk (*) represents significant differences (P < 0.05) between treated and control plots.*

**Supplementary Table S7** Detailed site information for studies selected in the meta-analysis.

| Reference | Location | Mein vegetation types | Cultivated age (yr) | Peat condition | Experimental mode | Annual mean precipitation (mm) | Annual mean temperature (°C) |
| --- | --- | --- | --- | --- | --- | --- | --- |
| Bonnett et al., 2017 | Geltsdale National Nature Reserve, UK | *Calluna vulgaris* L., *Eriophorum* spp., *S. capillifolium*, *S. cuspidatum*, *S. papillosum* | Long-term | Yes | Field | NA | NA |
| Burke et al. 1999 | the Donnelley Wildlife Management Area near Green Pond, S.C. | *Quercus alba* L. | Long-term | Yes | Field | NA | NA |
| Dieleman et al., 2016 | White River, Ontario, Canada | NA | 0.3-1.5 | Yes | Laboratory | NA | NA |
| Domínguez et al., 2016 | NE Wales, UK | *Calluna vulgaris* L. | Long-term | No | Field | 302 | 8.2 |
| Ellis et al., 2009 | the Migneint Valley, North Wales, UK | *Sphagnum* | 0.25 | Yes | Field | NA | NA |
| Fenner et al., 2020 | Migneint region, UK  Cors Goch, UK | *Sphagnum* | Long-term | Yes | Field | NA | NA |
| Fenner et al., 2005 | Cerrig-yr-Wyn in the Upper Wye catchment on Plynlimon, UK | *Sphagnum* | 0.3-0.5 | Yes | Field | NA | NA |
| Ge et al., 2020 | Baijianghe Peatland, China | *Carex lasiocarpa, Eriophorumvaginatum, Sphagnum palustre* | 0.2 | Yes | Laboratory | 761.5 | 2.8 |
| He et al., 2009 | Yichun, Heilongjiang, China | *Carex tristachya, Sphagnum* | 0.125 | Yes | Laboratory | NA | NA |
| Hribljan et al., 2014 | East of the Marsh Creek flooding | *Sphagnum Angustifolium Chamaedaphne calyculata* | Long-term | Yes | Field | 810 | 5.1 |
| Hribljan et al., 2017 | East of the Marsh Creek flooding | *Sphagnum* | Long-term | Yes | Field | 810 | NA |
| Huang et al., 2003 | The Cooper River of South Carolina | *Egeria, Cabomba, Hydrilla Ludwigia* spp*., Eichhornia crassipes* | Long-term | No | Field | NA | NA |
| Kane et al., 2013 | The Alaska Peatland | *Sphagnum, Equisetum, Carex, Potentilla* | Long-term | Yes | Field | NA | NA |
| Kang et al., 2011 | A ‘Mandahan’ marsh area in Malaysia | Grass | Long-term | No | Field | NA | NA |
| Könönen et al., 2016 | An ombrotrophic lowland peatland in Central Kalimantan, Indonesia | *Dipterocarpaceae* sp. | Long-term | Yes | Field | 2540 ± 596 | 26.2 ± 0.3 |
| Könönen et al., 2018 | The upper parts of the Sabangau River catchment in Central Kalimantan, Indonesia. | *NA* | 0.5 | Yes | Field | 2540 ± 596 | 26.2 ± 0.3 |
| Kwon et al., 2013 | Central Kalimantan, Indonesia | *Combretocarpus rotundatus, Danser, Cratoxylum glaucum Korth* | Long-term and 0.08 | Yes | Field&Laboratory | NA | NA |
| Ling et al., 2009 | Heilongjiang province, northeast of China | *Sphagnum, Carex* | 0.7 | Yes | Laboratory | NA | 18 |
| Liu et al., 2019 | Zoige wetland | *Alpinemeadow, Alpine swamp* | Long-term | Yes | Field | 650~750 | 0.7~1.1 |
| Liu et al., 2021 | Hongyuan-Zoige region on the Tibetan Plateau and Dajiuhu in Hubei Province, China | *Carex muliensis, Batrachium bungee, Sphagnum palustre, Carex argyi* | Long-term | Yes | Field | NA | NA |
| Mastný et al., 2016 | Bohemian Forest National Park in the southern part of the Czech Republic | Norway spruce | Long-term | Yes | Field | 1200 | 4 |
| Minick et al., 2022 | Alligator River National Wildlife Refuge (ARNWR) in Dare County, North Carolina | Nyssa sylvatica, Nyssa biflora, *Taxodium distichum* | Long-term | No | Field | 1270 | 16.9 |
| Negassa et al., 2022 | The Trebel and Recknitz valleys of the Federal State of Mecklenburg-Western Pomerania, northeastern Germany | *Carex acutiformis, Epilobium hirsutum, Deschampsia caespitosa* | Long-term | Yes | Field | NA | NA |
| Peacock et al., 2015 | The Migneint blanket bog, North Wales, UK | *NA* | Long-term | Yes | Field | 2540 ± 596 | 26.2 ± 0.3 |
| Reiche et al., 2009 | The Lehstenbach catchment area located in the northern Fichtelgebirge region in east central Germany | *Carex canescens, Carex rostrata, Juncus effusus* | 0.125-0.187 | Yes | Field | 1163 | 5.3 |
| Shahariar et al., 2021 | Indian Head, Saskatchewan, Canad | *Salix dasyclados, Bromus madritensis, cultivated oats, Avena sativa* | 0.2 | No | Laboratory | 605 | NA |
| Shao et al., 2016 | Yichun, Heilongjiang, China | Wood | Long-term | Yes | Field | 630 | 0.4 |
| Song et al., 2007 | Korea | *Phragmites australis* | 0.08 | No | Laboratory | NA | NA |
| Sun et al., 2010 | Xiaoxing’an Mountains, Heilongjiang province in North-east China | *Larix gmelini, Vaccinium uliginosum, Sphagnum* | 0.06 | Yes | Laboratory | NA | NA |
| Szajdak et al., 2021 | Mukhrino | *Sphagnum, cotton grass, sedge* | Long-term | Yes | Field | NA | NA |
| Toberman et al., 2008 | Cerrig-yr-Wyn in the Upper Wye catchment, Plynlimon, Wales | *Sphagnum, Juncus communities* | 0.08-0.17 | Yes | Laboratory | NA | NA |
| Toberman et al., 2010 | The Lakkasuo mire complex | *Carex rostrata Stokes., C. lasiocarpa Ehrh.* | Long-term | Yes | Field | 709 | 5 |
| Urbanová et al., 2018 | Bohemian Forest, south-western Czech Republic | *Andromeda polifolia, Vaccinium uliginosum, Eriophorum vaginatum, Carex limosa* | Long-term | Yes | Field | 1100-1260 | 3.2-4 |
| Vo et al., 2013 | Ewha Womans University campus, Seoul, Korea | *Phragmites australis* | 0.42 | No | Field | 1235 | NA |
| Wan et al., 2008 | Heilongjiang, China | *Carex lasiocarpa, Deyeuxia angustifolia* | 0.17 | Yes | Field | 550-600 | 1.9 |
| Wanet al., 2013 | Heilongjiang, China | *Calamagrostis angustifolia, Carex lasiocarpa, Carex pseudocuraica* | 0.25 | Yes | Field | 550-600 | 1.9 |
| Wang et al., 2015 | Pocosin Lakes National Wildlife Refuge (PLNWR) in coastal North Carolina, USA | Shrubs | 0.42 | Yes | Laboratory | NA | NA |
| Wang et al., 2017 | Luanhaizi wetland | *Carex pamirensis* | Long-term | Yes | Field | NA | NA |
| Wang et al., 2021 | Dunhua, Jilin Province, China | *Carex schmidtii* | Long-term | Yes | Field | 550-630 | 2.5-3.6 |
| Wang et al., 2021 | Jingyu, Jilin Province, China | *Carexsp., Iris tectorum, Menyanthestri-foliate, Sphagnum palustre* | Long-term | Yes | Field | 630 | 2-3 |
| Wang et al., 2022 | Baishan, Jilin Province, China | *Carex limosa, Eriophorum vaginatum, Sphagnum girgensohniiruss* | Long-term | Yes | Field | 761.5 | 2.8 |
| Webster et al., 2014 | Turkey Lakes Watershed | *Caltha palustris* L.*, Carex trisperma Dewey, Sphagnum cuspidatum* | 0.07 | Yes | Laboratory | NA | NA |
| Wen et al., 2019 | UK | *NA* | 0.25 | Yes | Laboratory | 612 | 13 |
| Xiang et al., 2013 | The Dajiuhu sub-alpine wetland in Mt. Shennongjia, China | *Sphagnum* | 0.08 | Yes | Laboratory | NA | NA |
| Xu et al., 2021 | Changbai Mountains, Jilin Province, Northeast China | *Carex rostrate* | Long-term | Yes | Field | 761.5 | 2.8 |
| Yan et al., 2020 | Zoige on the northeast edge of the Qinghai- Tibet Plateau | *Carex meyeriana, Koeleria tibetica, Carex muliensis* | 0.08 | Yes | Field | 650-750 | −1-3.3 |
| Yan et al., 2021 | Zoige on the northeast edge of the Qinghai- Tibet Plateau | *Koeleria tibetica, Carex meyeriana, Carex muliensis* | Long-term | Yes | Field | 650-750 | −1-3.3 |
| Yule et al., 2018 | Mulu National Park, Sarawak, East Malaysia | Macaranga | 0.25 | Yes | Field | NA | NA |
| Zeng et al., 2022 | Riganqiao, China | *Carex muliensis, Caltha palustris, Equisetum ramosissimum* | Long-term | Yes | Field | 860.8 | 2.9 |
| Zhang et al., 2018 | The Poyang Lake Wetland, Jiangxi Province, China | *Carex cinerascens, Phragmites australis, Triarrhena sacchariflora* | 0.42 | Yes | Field | 1680 | 17.5 |
| Zhang et al., 2019 | Baijainghe, Jilin, China | *Carexsp. Iris tectorum*  *Menyanthestri-foliate* | Long-term | Yes | Field | 761.5 | 2.8 |
| Zhang et al., 2019 | The dish lake of Nanji Wetland National Nature Reserve of Poyang Lake wetland. | *Carex. cinerascens Kükenth, Triarrhena lutarioriparia,* | Long-term | No | Field | 1358-1823 | 4.5 |
| Zhao et al., 2022 | Tonghua, China | *Carex schmidtii, Juncus effusus, Echinochloa crusgali* | Long-term | Yes | Field | NA | NA |
| Zhu et al., 2021 | The Dongting Lake Station for Wetland Ecosystem Research | *Carex brevicuspis* | 0.75 | No | Laboratory | NA | NA |

*NA represents unclear information.*

**References list for Table S5**

Deng, L., Peng, C.H., Kim, D., Li, J.W., Liu, Y.L., Hai, X.Y., Liu, Q.Y., Huang, C.B., Shangguan, Z.P., Kuzyakov, Y., 2021. Drought effects on soil carbon and nitrogen dynamics in global natural ecosystems. Earth-Science Reviews 214, 103501.

Gao, D.C., Bai, E., Li, M.H., Zhao, C.H., Yu, K.L., Hagedorn, F., 2020. Responses of soil nitrogen and phosphorus cycling to drying and rewetting cycles: A meta-analysis. Soil Biology and Biochemistry 148, 107896.

Margalef, O., Sardans, J., Maspons, J., Molowny-Horas R., Fernández-Martínez M., Janssens, I.A., Richter, A., Ciais, P., Obersteiner, M., Peñuelas, J., 2021. The effect of global change on soil phosphatase activity. Global Change Biology 00, 1-15.

Ren, C.J., Zhao, F.Z., Shi, Z., Chen, J., Han, X.H., Yang, G.H., Feng, Y.Z., Ren, G.H., 2017. Differential responses of soil microbial biomass and carbon-degrading enzyme activities to altered precipitation. Soil Biology and Biochemistry 115, 1-10.

Sun, Y., Liao, J.H., Zou, X.M., Xu, X.A., Yang, J.Y., Chen, H.Y.H., Ruan, H.H., 2020. Coherent responses of terrestrial C:N stoichiometry to drought across plants, soil, and microorganisms in forests and grasslands. Agricultural and Forest Meteorology 292-293, 108104.

Xiao, W., Chen, X., Jing, X., Zhu, B., 2018. A meta-analysis of soil extracellular enzyme activities in response to global change. Soil Biology and Biochemistry 123, 21-32.

**References list for Table S7 (there were 55 publications used in our meta-analysis)**

Bonnett, S.A.F., Maltby, E., Freeman, C., 2017. Hydrological legacy determines the type of enzyme inhibition in a peatlands chronosequence. Scientific Reports 7, 9948.

Burke, M.K., Lockaby, B.G. & Conner, W.H., 1999. Aboveground production and nutrient circulation along a flooding gradient in a south Carolina coastal plain forest. Canadian Journal of Forest Research 29, 1402-1418.

Dieleman, C.M., Branfireun, B.A., McLaughlin, J.W., et al., 2016. Enhanced carbon release under future climate conditions in a peatland mesocosm experiment: the role of phenolic compounds. Plant and Soil 400, 81-91.

Domínguez, M.T., Holthof, E., Smith, A.R., et al., 2017. Contrasting response of summer soil respiration and enzyme activities to long-term warming and drought in a wet shrubland (NE Wales, UK). Applied Soil Ecology 110, 151-155.

Ellis, T., Hill, P.W., Fenner, N., et al., 2009. The interactive effects of elevated carbon dioxide and water table draw-down on carbon cycling in a Welsh ombrotrophic bog. Ecological Engineering 35, 978-986.

Fenner, N., Freeman, C., 2020. Woody litter protects peat carbon stocks during drought. Nature Climate Change 10, 363.

Fenner, N., Freeman, C., Reynolds, B., 2005. Hydrological effects on the diversity of phenolic degrading bacteria in a peatland: implications for carbon cycling. Soil Biology & Biochemistry 37, 1277-1287.

Ge, L.M., Li, T., Yuan, X., et al., 2020. Simulation experiment on enzyme activity and carbon cycle of peat under short-term drainage. Wetland Science 18, 730-739.

He, L., Xiang, W., Sun, X.T., et al., 2009. The responses of enzyme activities to temperature and water level changes and the relationship between enzyme activity and CO_2_ emission flux in peatlands: A case study in Xiao'Xing'An'Ling mountain area. Ecology and Environmental Sciences 18, 2326-2333.

Hribljan, J.A., Kane, E.S., Pypker, T.G., et al., 2014. The effect of long-term water table manipulations on dissolved organic carbon dynamics in a poor fen peatland. Journal of Geophysical research-Biogeosciences 119, 577-595.

Hribljan, J.A., Kane, E.S., Chimner, R.A., 2017. Implications of altered hydrology for substrate quality and trace gas production in a poor fen peatland. Soil Science Society of America Journal 81, 633-646.

Huang, X.Q., Morris, J.T., 2003. Trends in phosphatase activity along a successional gradient of tidal freshwater marshes on the Cooper River, South Carolina. Estuaries 26, 1281-1290.

Kane, E.S., Chivers, M.R., Turetsky, M.R., et al., 2013. Response of anaerobic carbon cycling to water table manipulation in an Alaskan rich fen. Soil Biology & Biochemistry 58, 50-60.

Kang, H., Lee, S., Park, J., 2011. Microbial enzyme activities in tropical marsh soils with different water regime. Biology, Environment and Chemistry 151-153.

Könönen, M., Jauhiainen, J., Laiho, R., et al., 2016. Land use increases the recalcitrance of tropical peat. Wetlands ecology and Management 24, 717-731.

Könönen, M., Jauhiainen, J., Straková, P., et al., 2018. Deforested and drained tropical peatland sites show poorer peat substrate quality and lower microbial biomass and activity than unmanaged swamp forest. Soil Biology & Biochemistry 123, 229-241.

Kwon, M.J., Haraguchi, A., Kang, H., 2017. Long-term water regime differentiates changes in decomposition and microbial properties in tropical peat soils exposed to the short-term drought. Soil Biology & Biochemistry 60, 33-44.

Li, T., Ge, L.M., Huang, J.J., et al., 2020. Contrasting responses of soil exoenzymatic interactions and the dissociated carbon transformation to short- and long-term drainage in a minerotrophic peatland. Geoderma 377, 114585.

Ling, H., Wu, X., Sun, XT., 2009. Effects of temperature and water level changes on enzyme activities in two typical peatlands: implications for the responses of carbon cycling in peatland to global climate change. International Conference on Environmental Science & Information Application Technology IEEE Computer Society, 18-22.

Liu, Y., Wang, X.Q., Shen, D.J., et al., 2019. Soil enzymatic activities dynamics along a moisture gradient in alpine wetland in western Sichuan Province. Journal of Sichuan Agricultural University 37, 517-524.

Liu, C.Z., Wang, S.M., Zhu, E.X., et al., 2021. Long-term drainage induces divergent changes of soil organic carbon contents but enhances microbial carbon accumulation in fen and bog. Geoderma 404.

Mastny, J., Urbanová, Z., Kastovská, E., et al., 2016. Soil organic matter quality and microbial activities in spruce swamp forests affected by drainage and water regime restoration. Soil Use and Management 32, 200-209.

Minick, K.J., Aguilos, M., Li, X.F., et al., 2022. Effects of spatial variability and drainage on extracellular enzyme activity in coastal freshwater forested wetlands of eastern North Carolina, USA. Forests 13.

Negassa, W., Baum, C., Beyer, F., et al., 2022. Spatial variability of selected soil properties in long-term drained and restored peatlands. Frontiers in Environmental Science 10.

Peacock, M., Jones, T.G., Airey, B., et al., 2015. The effect of peatland drainage and rewetting (ditch blocking) on extracellular enzyme activities and water chemistry. Soil Use and Management 31, 67-76.

Reiche, M., Hädrich, A., Lischeid, G., et al., 2009. Impact of manipulated drought and heavy rainfall events on peat mineralization processes and source-sink functions of an acidic fen. Journal of Geophysical research- Biogeosciences 114.

Shahariar, S., Helgason, B., Soolanayakanahally, R., et al., 2021. Soil enzyme activity as affected by land-use, salinity, and groundwater fluctuations in wetland soils of the prairie pothole region. Wetlands 41.

Shao, Z.R., Zhao, G.Y., Zang, S.Y., et al., 2016. Effect of drainage afforestation wetland on soil enzyme activity and nitrogen content in xiaoxing'an mountains. Environment Engineering 34, 122-126.

Song, K.Y., Zoh, K.D., Kang, H., 2007. Release of phosphate in a wetland by changes in hydrological regime. Science of the Total Environment 380, 13-18.

Sun, X.T., Xiang, W., He, L., et al., 2010. Impacts of hydrological conditions on enzyme activities and phenolic concentrations in peatland soil: An experimental simulation. Frontiers of Earth Science 4, 463-470.

Szajdak, L.W., Meysner, T., Szczepanski, M., et al., 2021. Enzymatic activity as new moorsh-forming process indicators of peatlands. Agronomy-Basel 11.

Toberman, H., Freeman, C., Artz, R.R.E., et al., 2008. Impeded drainage stimulates extracellular phenol oxidase activity in riparian peat cores. Soil Use and Management 24, 357-365.

Toberman, H., Laiho, R., Evans, C.D., et al., 2010. Long-term drainage for forestry inhibits extracellular phenol oxidase activity in Finnish boreal mire peat. European Journal of Science 61, 950-957.

Urbanová Z., Straková P., Kaštovská E., 2018. Response of peat biogeochemistry and soil organic matter quality to rewetting in bogs and spruce swamp forests. European Journal of Soil Biology 85,12-22.

Vo, N.X.G., Kang, H., 2013. Regulation of soil enzyme activities in constructed wetlands under a short-term drying period. Chemistry and Ecology 29, 146-165.

Wan, Z.M., Song, C.C., Guo, Y.D., et al., 2008. Effects of water gradient on soil enzyme activity and active organic carbon composition under *Carex lasiocarpa* marsh. Acta Ecologica Sinica 28, 5980-5986.

Wan, Z.M., 2013. Effects of water level on CO_2_ and CH_4_ flux and soil microbial activity in Calamagrostis angustifolia marsh. Ecology and Environmental Sciences 22, 465-468.

Wang, H.J., Richardson, C.J., Ho, M.C., 2015. Dual controls on carbon loss during drought in peatlands. Nature Climate Change 5, 584-587.

Wang, Y.Y., Wang, H., He, J.S., et al., 2017. Iron-mediated soil carbon response to water-table decline in an alpine wetland. Nature Communications 8,15972.

Wang, M., Han, Y.Y., Xu, Z.W., et al., 2021. Hummock-hollow microtopography affects soil enzyme activity by creating environmental heterogeneity in the sedge-dominated peatlands of the Changbai Mountains, China. Ecological Indicators 121, 107187.

Wang, Y.N., Xu, Z.W., Wang, S.Z., 2021. Concentrations of active organic carbon components in soils in Baijianghe natural and drained peat bogs and their influencing factors. Wetland Science 19, 691-701.

Wang, Y.T., Xu, Z.W., Sun, D.J., et al., 2022. Effect of water table restoration on soil enzyme activities of the drained peatland. Chinese Journal of Ecology 41, 1940-1947.

Webster, K.L., Creed, I.F., Malakoff, T., et al., 2014. Potential vulnerability of deep carbon deposits of forested swamps to drought. Soil Science Society of America Journal 78, 1097-1107.

Wen, Y., Zang H.D., Ma, Q.X., et al., 2003. Is the ‘enzyme latch’ or ‘iron gate’ the key to protecting soil organic carbon in peatlands? Geoderma 349,107-113.

Xiang, W., Wan, X., Yan, S., et al., 2013 Inhibitory effects of drought induced acidification on phenol oxidase activities in Sphagnum-dominated peatland. Biogeochemistry 116, 293-301.

Xu, ZW., Wang, S.Z., Wang, Z.C., et al., 2021. Effect of drainage on microbial enzyme activities and communities dependent on depth in peatland soil. Biogeochemistry 155, 323-341.

Yan, Z.Q., Li, Y., Wu, H.D., et al., 2020. Different responses of soil hydrolases and oxidases to extreme drought in an alpine peatland on the Qinghai-Tibet Plateau, China. European Journal of Soil Biology 99, 103195.

Yan, Z.Q., Kang, E.Z., Zhang, K.R., et al., 2021. Plant and soil enzyme activities regulate CO_2_ efflux in alpine peatlands after 5 years of simulated extreme drought. Frontiers in Plant Science 12.

Yule, C.M., Lim, Y.Y., Lim, T.Y., 2018. Recycling of phenolic compounds in Borneo’s tropical peat swamp forests. Carbon Balance and Management 13.

Zeng, J., Chen, H., Liu, J.L., 2022. The decrease of peatland water table on the Qinghai-Tibet Plateau caused the increase of soil phenolic substances and vegetation biomass which promoted the accumulation of soil carbon. Acta Ecologica Sinica 42, 625-634.

Zhang, G.S., Yu, X.B., Xu, J., et al., 2018. Effects of environmental variation on stable isotope abundances during typical seasonal floodplain dry season litter decomposition. Science of The Total Environment 630, 1205-1215.

Zhang, Y.P., Liu, S.N., Xu, Z.W., et al., 2019. Soil enzyme activities in natural and drainage peat bogs in Baijianghe of Changbai Mountains. Wetland Science 17, 445-452.

Zhang, Q.J., Zhang, G.S., Yu, X.B., et al., 2019. Effect of ground water level on the release of carbon, nitrogen and phosphorus during decomposition of Carex. cinerascens Kükenth in the typical seasonal floodplain in dry season. Journal of Freshwater Ecology 34, 305-322.

Zhao, J., Duan, L.L., Wang, M., et al., 2022. Effects of hydrological managements on soil enzyme activities during peatland restoration in the Changbai Mountains. Chinese Journal of Ecology 41, 948-954.

Zhu, L.L., Deng, Z.M., Xie, Y.H., et al., 2021. Factors controlling *Carex brevicuspis* leaf litter decomposition and its contribution to surface soil organic carbon pool at different water levels. Biogeosciences 18, 1-11.
